# Interactions between plant‐beneficial microorganisms in a consortium: *Streptomyces microflavus* and *Trichoderma harzianum*

**DOI:** 10.1111/1751-7915.14311

**Published:** 2023-07-18

**Authors:** Maria Isabella Prigigallo, Alessia Staropoli, Francesco Vinale, Giovanni Bubici

**Affiliations:** ^1^ Istituto per la Protezione Sostenibile delle Piante Consiglio Nazionale delle Ricerche Bari Italy; ^2^ Istituto per la Protezione Sostenibile delle Piante Consiglio Nazionale delle Ricerche Portici Italy; ^3^ Dipartimento di Agraria Università degli Studi di Napoli Federico II Portici Italy; ^4^ Dipartimento di Medicina Veterinaria e Produzioni Animali Università degli Studi di Napoli Federico II Naples Italy

## Abstract

The construction of microbial consortia is challenging due to many variables to be controlled, including the cross‐compatibility of the selected strains and their additive or synergistic effects on plants. In this work, we investigated the interactions in vitro, in planta, and at the molecular level of two elite biological control agents (BCAs), that is *Streptomyces microflavus* strain AtB‐42 and *Trichoderma harzianum* strain M10, to understand their attitude to cooperate in a consortium. In vitro, we observed a strong cross‐antagonism between AtB‐42 and M10 in agar plates due to diffusible metabolites and volatile organic compounds. In liquid co‐cultures, M10 hindered the growth of AtB‐42 very likely because of secondary metabolites and strong competition for the nutrients. The interaction in the co‐culture induced extensive transcriptional reprogramming in both strains, especially in the pathways related to ribosomes, protein synthesis, and oxidoreductase activity, suggesting that each strain recognized the counterpart and activated its defence responses. The metabolome of both strains was also significantly affected. In contrast, in the soil, M10 growth was partially contrasted by AtB‐42. The roots of tomato seedlings inoculated with the consortium appeared smaller than the control and single‐strain‐inoculated plants, indicating that plants diverted some energy from the development to defence activation, as evidenced by the leaf transcriptome. The consortium induced a stronger transcriptional change compared to the single inoculants, as demonstrated by a higher number of differentially expressed genes. Although the cross‐antagonism observed in vitro, the two strains exerted a synergistic effect on tomato seedlings by inducing resistance responses stronger than the single inoculants. Our observations pose a question on the usefulness of the sole in vitro assays for selecting BCAs to construct a consortium. In vivo experiments should be preferred, and transcriptomics may greatly help to elucidate the activity of the BCAs beyond the phenotypic effects on the plant.

## Graphical abstract

The construction of microbial consortia is challenging due to many variables to be controlled, including the cross‐compatibility of the selected strains and their additive or synergistic effects on plants. In this work, we investigated the interactions in vitro, in planta, and at the molecular level of two elite biological control agents (BCAs), i.e., *Streptomyces microflavus* strain AtB‐42 and *Trichoderma harzianum* strain M10, to understand their attitude to cooperate in a consortium. Microorganisms, including BCAs, compete against every other microorganism to preserve the species and their niche. Although we observed a cross‐antagonism in vitro, these two strains exerted synergistic effects in vivo on tomatoes. Whenever possible, hence, it would be more advantageous to study the microorganisms in vivo. Finally, the analysis of the plant transcriptome represented a valuable tool for interpreting the phenotypic effects of the BCAs on the crop.

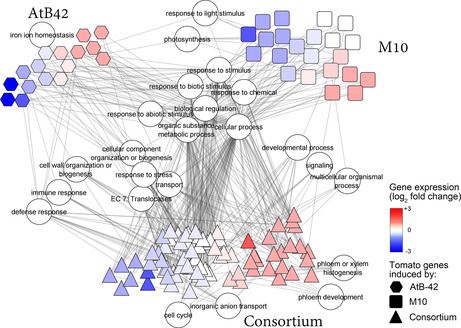

## INTRODUCTION

In recent years, microbe‐assisted crop production approaches have attracted the interest of the scientific community because of their safer use and environmental friendliness compared to conventional agriculture. Plant‐beneficial microorganisms (or their metabolites), with their ability to improve crop productivity and resilience, offer a multitude of chances to ameliorate agricultural sustainability (Arif et al., 2020; Hohmann et al., 2020). Furthermore, there has been an increasing trend of the use of artificial microbial consortia rather than single strains with the aim to deploy complementary and synergistic activities of their components, though formulations with more than three species are still limited (Morales‐García et al., 2019). Typically, a microbial consortium is composed of compatible strains with different modes of action (Prigigallo et al., 2022). However, the development and optimization of a microbial consortium need care, especially regarding its ability to survive and be biologically active under diverse environmental conditions, thus meaning that a mere combination of microbes can yield unexpected results (Mikesková et al., 2012). Recent research has demonstrated how the in vitro study of the interactions among antagonists of phytopathogens is one of the principles for designing an effective synthetic microbial community (SynCom; Prigigallo et al., 2022). The poor understanding of SynCom functioning as a whole might result in unexpectedly low effectiveness. Moreover, reductionist research approaches based on the study of one‐to‐one microbial interactions significantly reduce the quality of the information about the mechanistic basis of microbial communities (Pozo et al., 2021). Holistic approaches such as transcriptomics, proteomics, metabolomics, etc. provide deeper and wider insights into the intricate interaction network of microbial communities, both within them and with other microbes (e.g., phytopathogens), the plants and the environment (Palmieri et al., 2022). Agler et al. (2016) have envisioned that microorganisms work as ‘keystone nodes’, ‘hub nodes’ or ‘edge nodes’ based on their role in a community. Keystone nodes are greatly responsible for the community structure, as without them the community dynamics change. Hub nodes are highly connected to the network, transmit to other nodes, especially the neighbourhoods, the signals perceived (e.g., abiotic factors) and thus shape the community structure and functioning. Those authors have proposed to focus on keystone and hub taxa to manipulate microbial communities more efficiently. A similar concept has also been envisaged by Toju et al. (2020), who suggested focusing on ‘functional core microbiomes’, viz. species groups with potentially larger contributions to ecosystem‐level functions. Fungal networks have appeared to be more resilient than bacteria to environmental factors (de Vries et al., 2018), thus probably contributing to the stability of the overall microbiota (Pozo et al., 2021). In practice, the use of microbial consortia is still challenging because results cannot be easily predicted as with chemical pesticides. Furthermore, the current European legislation [e.g., Commission Regulations (EU) 283/2013 and 284/2013] makes the registration of microbial biological control agents (BCAs) expensive and highly demanding due to the huge scientific data requirements and risk assessment (Liebenberg & Huber, 2022). Also, several beneficial microorganisms exert both biocontrol and growth promotion activities, while plant protection products and biostimulants are currently regulated under different laws (Woo & Pepe, 2018).

The combination of fungi and bacteria offers great potential for microbe‐assisted crop cultivation. Several studies have been done on the combined use of *Trichoderma* spp. and bacteria, mainly *Bacillus* spp. and *Pseudomonas* spp., for biocontrol or plant growth promotion on various crops (Hafiz et al., 2022; Izquierdo‐Garcia et al., 2020; Poveda & Eugui, 2022). Moreover, the combination of metabolites of these microorganisms has been demonstrated to be synergistic. For example, cell wall degrading enzymes produced by *Trichoderma* spp. have increased the sensitivity of several plant pathogens to syringotoxins and syringomycins purified from *P. syringae* (Woo et al., 2002). The combination of *Trichoderma* spp. with *Streptomyces* spp. has been poorly studied. The application of *Trichoderma* spp. in agriculture as BCAs and/or plant growth‐promoting microorganisms has been documented since the 1930s (Lorito et al., 2010; Weindling & Fawcett, 1936), and a huge number of *Trichoderma*‐based products are currently on the market (Woo et al., 2023). The metabolome of this fungal genus is incredibly complex and can impact the phytopathogens directly through antagonism or indirectly by triggering the defence system of the host plant (Reino et al., 2008; Vinale et al., 2012). Moreover, plant growth promotion represents an important activity of *Trichoderma* species (El Enshasy et al., 2020; Harman et al., 2004), though it has been more commonly ascribed to bacteria (Morales‐García et al., 2019). *Pseudomonas*, *Bacillus*, *Rhizobium*, *Azospirillum*, and a few others are the most commonly used bacterial genera in agriculture (Morales‐García et al., 2019). Streptomycetes have been studied less than other bacteria as BCAs and very few products are commercialized for agricultural use (Bubici, 2018). They are the largest taxon of antibiotic producers in the microbial world and use their antibiotics along with a plethora of secondary metabolites and hydrolytic enzymes to compete against other microbes, including plant pathogens (Bubici, 2018). Their complex metabolome might have limited the registration for agricultural use, while they have received much more interest for industrial and biotechnological purposes (Chater, 2016).

Mixing beneficial microorganisms of different taxa is often preferred to obtain additive effects or even the synergism of their modes of action. On the other hand, the production costs of microbial consortia should be carefully assessed as they increase with the number of strains included in the consortia. The general trend in combining microbial strains underlies the assumption that beneficial microorganisms are such in absolute, that is not true. Nevertheless, due to the inconsistent results of BCAs often observed across environments and seasons, and to the increasing attention to microbiomes rather than single strains, various strategies for designing microbial consortia are being proposed (Kehe et al., 2019; Toju et al., 2020). In recent research, we observed that a three‐strain SynCom was more effective than a 44‐strain one against the *Fusarium* wilt of bananas very likely because of cross‐antagonism among strains (Prigigallo et al., 2022). It was shown that beneficial microbes, even if selected for the desired trait (e.g., antagonism against a certain pathogen), compete with other microbes in the environment to protect their niche. In other words, we believe that synergistic activities among beneficial microorganisms are an exception rather than a rule in nature. In this view, here we investigate the interactions between two well‐known BCAs, that is *Streptomyces microflavus* strain AtB‐42 and *Trichoderma harzianum* strain M10, taxonomically distant and with elite modes of action potentially different. The former strain acts mainly by antibiosis (Bubici et al., 2013) and the latter is a plant growth promoter, though it can also induce plant defence (Manganiello et al., 2018). Both strains can survive in the soil even in the absence of plants. Due to their effectiveness, different main modes of action and taxonomical distance, they could be good candidates for a consortium of BCAs. Here, we show that although the cross‐antagonism observed in vitro, these two strains had synergistic effects in vivo on tomato plants.

## EXPERIMENTAL PROCEDURES

Two elite microbial strains with diverse modes of action were used in this study: *S. microflavus* strain AtB‐42 and *T. harzianum* strain M10. The strain AtB‐42 has been previously selected based on several in vitro and in planta assays and has been proven to be effective in the field against the corky root of tomato, caused by *Pyrenochaeta lycopersici* (Bubici et al., 2013; Colella et al., 2001). It has a strong antagonistic activity against several plant pathogens (Bubici, 2006). *Trichoderma harzianum* strain M10, a producer of a metabolite with antifungal properties namely harzianic acid (Vinale et al., 2009), has shown a major plant growth promotion activity (Pascale et al., 2017), besides other properties such as the iron chelation and induction of plant defence responses (Manganiello et al., 2018).

To evaluate the interactions between these two strains, several in vitro (both in agar media and liquid co‐cultures) and in vivo (in sterilized soil) experiments were conducted.

### Interactions in Petri plates

The capability of agar‐diffusible metabolites (DM) and volatile organic compounds (VOCs) produced by AtB‐42 and M10 to affect the growth of each other was evaluated. The cellophane‐agar (CA) technique (Prigigallo et al., 2022) and the overlapping plate (OP) method (Prigigallo et al., 2021) were used to evaluate the effects of DM and VOCs, respectively. In addition, the simultaneous activity of both DM and VOCs produced by AtB‐42 against M10 was evaluated by the dual culture assay (Bubici et al., 2013). This method was not used for M10 because of its rapid growth. The assays were conducted in triplicate (three plates) at 27°C in the dark.

### Interactions in liquid co‐cultures

The interaction between AtB‐42 and M10 was also studied by determining their population kinetics over time in the co‐culture compared to the single culture. The experiment was carried out in 50 mL tubes (Corning™ Falcon™, Fisher Scientific Italia) containing 20 mL of potato dextrose broth (PDB, Condalab). AtB‐42 and M10 inocula were prepared by washing with sterile distilled water 14‐day‐ and 7‐day‐PDA plates, respectively. The concentration of spores (AtB‐42) or conidia (M10) was estimated using a Thoma cell counting chamber (BRAND® counting chamber BLAUBRAND®, Merck KGaA). The spores/conidia suspensions were inoculated into single‐ and co‐cultures to reach certain initial concentrations of AtB‐42 and M10. Two independent experiments (A1 and A2) were conducted using diverse initial concentrations of AtB‐42 and M10: 10^3^ spores mL^−1^ of AtB‐42 and 10^2^ conidia mL^−1^ of M10 in Experiment A1, while 10^5^ spores mL^−1^ of AtB‐42 and 10^4^ conidia mL^−1^ of M10 in Experiment A2. In both experiments, AtB‐42 was inoculated 3 days before M10 and at higher concentrations, either in single or co‐cultures, because of its slower growth (timing determined on preliminary assays; data not shown). The liquid cultures were prepared in triplicate, that is three tubes per culture, for a total of nine tubes and incubated at 27°C in an orbital shaker at 250 rpm. The growth of AtB‐42 and M10 was measured at several time points, that is days from inoculation of the co‐culture (*dfi*) with M10: −3, 0, 4, 7, 11 and 14 *dfi*. The microbial growth was determined by the dilution plate technique (90 mm Petri plates) on semi‐selective agar media: starch‐casein‐KNO_3_ (SCPN) agar for AtB‐42 (Küster & Williams, 1964) and *T. harzianum*‐selective medium (THSM) for M10 (Williams et al., 2003). After 7 days of incubation at 27°C in the dark, the colonies in the plates were counted.

Experiment A2 was also used for transcriptomics (RNA‐Seq) and metabolomics analyses. For RNA extraction, 2 mL cultures were taken three *dfi* (i.e. a time point during the decreasing phase of AtB‐42 growth) from each of the nine tubes and centrifuged at 16,000× *g* for 15 min. The pellet was immediately re‐suspended in the lysis buffer of the Quick‐RNA Fungal/Bacterial Miniprep Kit (Zymo Research) and RNA was extracted according to the manufacturer's instructions. Then, RNA was subjected to DNase digestion using TURBO DNA‐free™ Kit (Thermo Fisher Scientific), quantified by NanoDrop 1000 (Thermo Fisher Scientific), and its quality was checked by gel electrophoresis before sending it to Macrogen Europe for the library preparation and next‐generation sequencing. Before library preparation, RNA samples from AtB‐42 and the co‐cultures were subjected to bacterial rRNA depletion using the Ribo‐Zero Plus Kit (Illumina). Libraries of the AtB‐42 samples were prepared using the Illumina® Stranded Total RNA Prep, while those of M10 and the co‐cultures using Illumina® Stranded Total RNA Prep Gold. NovaSeq 6000 system (Illumina) was used for sequencing 30 million reads per sample with 100 bp in paired‐end mode. Data were deposited in GenBank with the BioProject ID PRJNA954200.

For the metabolomic analysis, liquid single and co‐cultures at 14 *dfi* were filtered through a Miracloth filter paper (Millipore®) at atmospheric pressure and directly analysed using a quadrupole‐time of flight (Q‐TOF) mass spectrometer (Agilent Technologies) coupled to an Agilent HP 1260 Infinity Series liquid chromatograph (Agilent Technologies). Separation was performed using a Zorbax Extend C‐18 column (4.6 × 50 mm, 3.5 μm, Agilent Technologies), held at 37°C. Elution gradient consisted of 0.1% (v/v) formic acid in water (A) and 0.1% (v/v) formic acid in acetonitrile (B) and the program was the following: from 5% to 100% B in 6 min, isocratic at 100% B for 2 min; from 100% to 5% B in 2 min. Equilibration time was 2 min; flow rate was 0.4 mL min^−1^; injection volume was 7 μL. All spectrometric parameters were set according to Vinale et al. (2020) by using the Agilent MassHunter Data Acquisition Software, rev. B.05.01 (Agilent Technologies).

### Interactions in sterilized soil

The interaction between AtB‐42 and M10 was also studied in vivo, that is the soil planted with tomatoes (*Solanum lycopersicum* L.; Experiment B1). Soil was Rodhic, chromic, calcic luvisol (‘terra rossa’ soil with about 1% organic carbon). Heat‐sterilized soil was preferred to the natural field soil to avoid possible and unpredictable interference of the natural microbiota with the inoculated strains. Heat‐sterilization was conducted as follows: 20 kg soil batches were wetted at the field capacity, placed in steel trays and heated at 80°C (temperature measured in the soil) for 24 h in a laboratory stove; then, the soil was transferred to clean containers, where it remained at least one week before use. The soil was inoculated with AtB‐42 (10^6^ spores g^−1^ of soil), M10 (10^4^ conidia g^−1^), their combination (consortium) or sterile distilled water (control). Inocula were prepared as described above. Due to the slower growth, AtB‐42 was inoculated with a higher inoculum and 3 days before M10, which was inoculated at transplanting. The soil was inoculated by drenching with 100 mL of inoculum per 1 L of soil and then mixed manually to ensure the uniformity of inoculum distribution. At transplanting, the soil was poured into 1.4 L‐plastic pots and 30 days‐old tomato seedlings cv. UC82 were used. The pots were arranged in a randomized complete block design with three replicates, each with three pots (one plant per pot) and maintained in a glasshouse under natural lighting (ca. 27/19 ± 3°C day night^−1^). Plants were irrigated as needed and fertilized once a week with Hoagland's solution (Hoagland & Arnon, 1950), while no phytosanitary treatments were made.

The soil population kinetics of both strains was determined using the aforementioned dilution plate technique and semi‐selective media at 0, 7, 14 and 28 days post‐transplanting (*dpt*). For this purpose, 5 g‐soil samples were taken at 5 cm depth from each pot. Samples of the three pots of each replicate were mixed and one sample per replicate was analysed (three plates per sample and dilution). After 7 days of incubation, colonies were counted and the microbial populations were expressed as colony‐forming units (CFUs) per dry weight (g) of soil (determined on a separate soil aliquot after drying at 70°C until constant weight).

Plant height was measured at 7, 14, 21 and 28 *dpt*, while shoot and root fresh weight (expressed as g plant^−1^) were measured at the end of the experiment, that is 28 *dpt*.

Fourteen days post‐transplanting, leaves were sampled for the RNA‐Seq analysis. The youngest fully expanded leaf of each plant was taken, and the leaves from the three plants of each replicate were pooled to have one sample per replicate. The RNA was extracted using Plant Total RNA Purification Mini Kit (Fisher Molecular Biology) and subjected to DNase digestion using TURBO DNA‐free™ Kit according to the manufacturer's instructions. After quantification with NanoDrop 1000 and the quality check by gel electrophoresis, RNA was sent to GenomiX4Life for library preparation and next‐generation sequencing. Libraries were constructed using the TruSeq® Stranded mRNA Library Prep and sequenced in paired‐end mode with 100 bp reads and 30 million reads (2 × 15) per sample (NovaSeq 6000; Illumina). Data were deposited in GenBank with the BioProject ID PRJNA954272.

Two more independent in vivo experiments (B2 and B3) were conducted with a slightly different inoculation protocol to confirm the effect of the strains on tomato development (Experiments B2 and B3). In these experiments, AtB‐42 (10^6^ spores g^−1^ of soil), M10 (10^4^ conidia g^−1^) or their combination (consortium) were inoculated simultaneously at transplanting, 7 and 14 *dpt*. In Experiment B2, plant height, shoot and root fresh weight were measured. In Experiment B3, plant height and normalized difference vegetation index (NDVI; measured by GreenSeeker handheld crop sensor; Trimble) were determined.

### Analysis of RNA‐Seq data

Data analysis was carried out using the Galaxy platform version 20.01 (Afgan et al., 2018) locally installed on a computer equipped with 16 core‐CPU and 64 GB RAM. Raw data (FASTQ file format) were subjected to quality check and filtering using FASTQC v. 0.72 + galaxy1 and Filter FASTQ tool v. 1.1.1 (Blankenberg et al., 2010), respectively: reads containing bases with a Phred quality score lower than 20 were discarded. Clean reads were mapped to the reference genomes using Bowtie2 2.5.0 + galaxy0 (Langmead & Salzberg, 2012) and counted using featureCounts 2.0.1 + galaxy2 (Liao et al., 2014). The publicly available sequences of *S. microflavus* strain DSM 40593 (Myronovskyi et al., 2013) [100% bootstrap clustering with AtB‐42 in a multilocus sequence analysis; Bubici et al., 2018], *T. harzianum* M10 v1.0 (Project ID: 1185310; Joint Genome Institute, Berkeley, CA, USA), and tomato SL4.0 (https://solgenomics.net) were used as reference genomes. Differentially expressed genes (DEGs) were identified by DESeq2 (Love et al., 2014) using a threshold of false discovery rate (FDR) below 0.05. The genomes were re‐annotated using Blast2GO 6.0.3 and the gene ontology (GO) enrichment analysis was performed based on Fisher's exact test (FDR < 0.05; Conesa et al., 2005). The antiSMASH 6.0 was used to annotate the genomes with the secondary metabolite biosynthetic gene clusters (Blin et al., 2021). Plots were generated using R 4.1.1 (ISBN 3‐900051‐07‐0; http://www.Rproject.org) and the ggplot2 3.4.0 package (Wickham et al., 2016) within RStudio 2021.09.0 build 351 (http://www.rstudio.com). The network chart was made with Cytoscape 3.9.1 (Shannon et al., 2003).

### Statistical analysis

Data from the biological experiments were subjected to the analysis of variance (ANOVA) after checking the assumptions for its validity using Shapiro–Wilk's test and Bartlett's test. Data of microbial population and gene expression were log‐transformed before ANOVA to fulfil the assumption for the parametric statistics. The *t*‐test (*p* < 0.05) was used to compare two samples (e.g., treated vs. control) and Tukey's test (*p* < 0.05) for the multiple comparisons of the means. These statistical analyses were conducted with R 4.1.1 and RStudio 2021.09.0 build 351.

Statistical analysis of the metabolomics dataset was performed using Mass Profiler Professional software, version 13.1.1 (Agilent Technologies). Data were subjected to principal component analyses (PCA) and Student *t*‐test (*p* < 0.05). Identification of the metabolites was done by searching spectrometric data in the literature and the KNApSAcK database (freely available at http://www.knapsackfamily.com/KNApSAcK). The identification was considered successful if the mass error was below 10 ppm.

## RESULTS

### AtB‐42 and M10 exert a reciprocal antagonism in Petri plates

**FIGURE 1:**
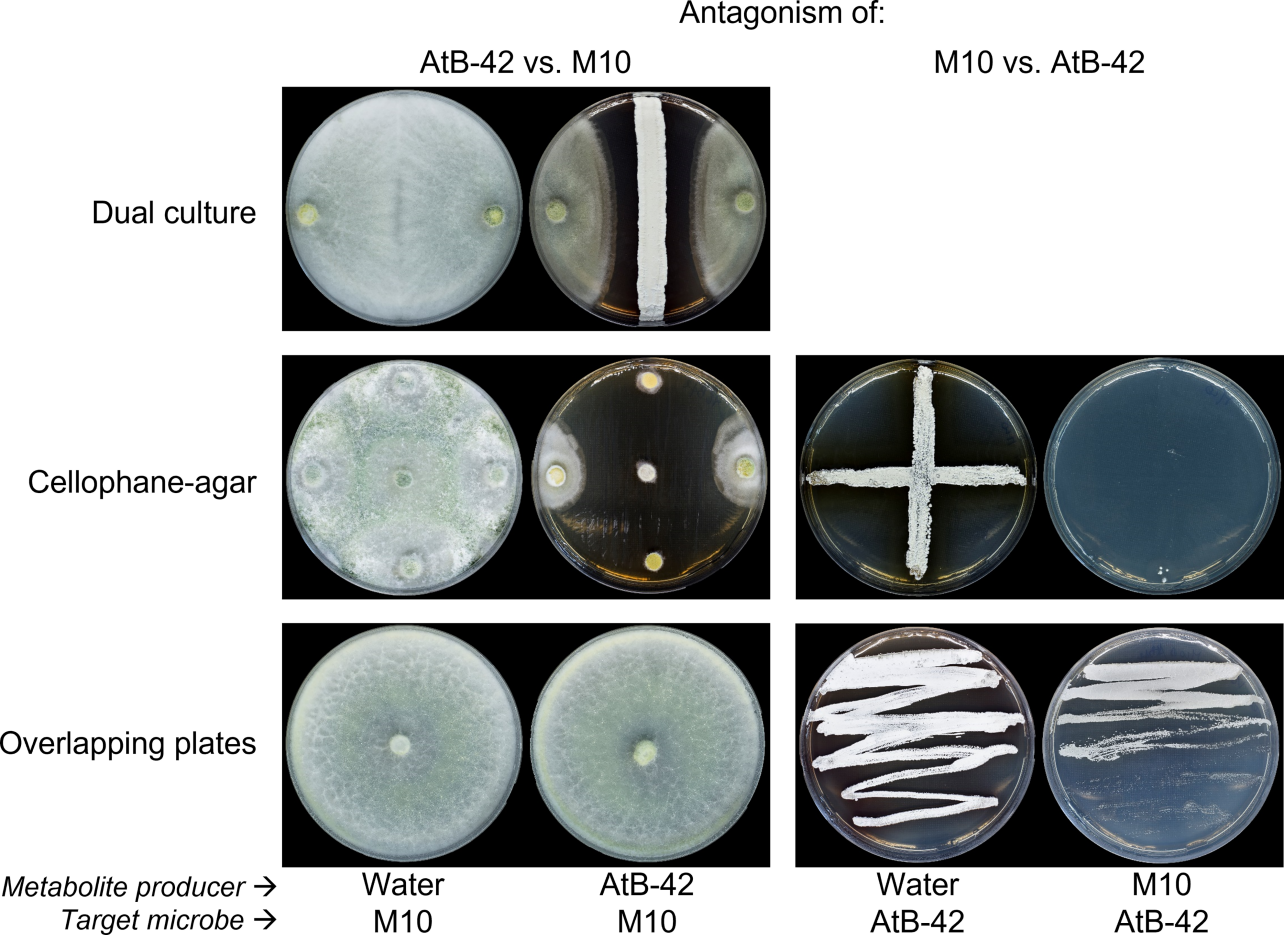
In vitro assays for the reciprocal antagonism of *Streptomyces microflavus* AtB‐42 and *Trichoderma harzianum* M10.

The effects of DMs and VOCs produced by AtB‐42 and M10 were evaluated by three types of in vitro assays (Figure 1). In the DC assay, which is useful to evaluate the effects of both DM and VOCs, AtB‐42 reduced M10 growth by ca. 50%. The reciprocal assay, viz. with M10 as a source of metabolites and AtB‐42 as a target strain, was not done because the fast growth of M10 and slow growth of AtB‐42 did not allow a reliable confrontation of the strains.

In the CA assay, which evaluates only the DMs, AtB‐42 hindered M10 growth in a dose‐dependent manner. In fact, no growth arose from the M10 agar plugs placed on the lane (one diameter of the plate) previously hosting the AtB‐42 streak, but it arose (though significantly reduced compared to control) from those placed at the edges of the orthogonal diameter, where the metabolites diffused through the agar medium following a decreasing concentration gradient. With the same method, M10's metabolites released in the agar medium completely inhibited AtB‐42 growth.

The OP assay, which can be used to evaluate the VOC effects only, showed that AtB‐42 did not produce VOCs toxic to M10, while the M10's volatilome partially reduced the growth of AtB‐42.

### M10 hinders AtB‐42 growth in liquid co‐cultures

**FIGURE 2:**
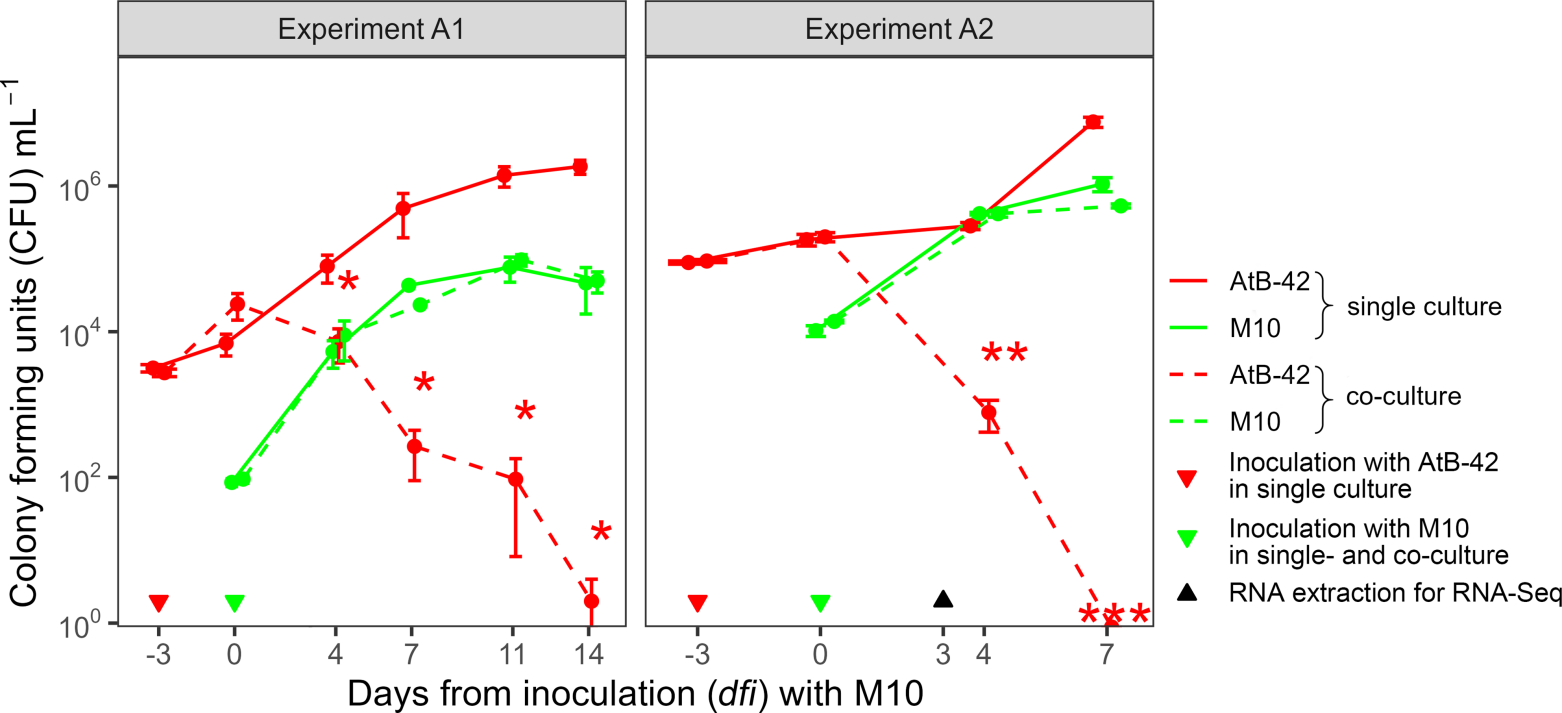
In vitro liquid co‐culture assays. Growth curves of *Streptomyces microflavus* AtB‐42, *Trichoderma harzianum* M10 in single cultures and co‐cultures. Experiments A1 and A2 differ for the initial inoculum concentrations. Error bars represent the standard error of the mean (*n* = 3). Asterisks indicate a significant difference in the co‐culture compared to the single culture according to Student's *t*‐test (*p < 0.05; **p < 0.01; ***p < 0.001).

The population kinetics of AtB‐42 and M10 was determined in liquid cultures (50 mL tubes with 20 mL LB broth) to assess their interactions in two experiments (A1 and A2) differing for the initial inoculum concentrations (Figure 2).

In Experiment A1, the M10 population in the single culture was 85 ± 10 CFUs·mL^−1^ at the inoculation time (0 *dfi*) and 4.7 ± 2.9 × 10^4^ CFUs·mL^−1^ at 14 *dfi*. Similarly, in the co‐culture, M10 grew from 95 ± 12 to 5 ± 1.6 × 10^4^ CFUs·mL^−1^, with no significant difference between the co‐culture and single culture at every time point. Single cultures of AtB‐42 were inoculated at −3 *dfi* with 3.2 ± 0.4 × 10^3^ CFUs·mL^−1^ and grew up to 1.9 ± 0.4 × 10^6^ CFUs·mL^−1^ at 14 *dfi*. In the co‐cultures, M10 was inoculated at 0 *dfi*, when AtB‐42 had 2.4 ± 0.9 × 10^4^ CFUs·mL^−1^. In these tubes, AtB‐42 stopped its growth and the population decreased progressively until 2 ± 1 CFUs·mL^−1^ with significant (*p* < 0.05) differences compared to single cultures.

Results observed in Experiment A2 were very similar to those obtained in Experiment A1, though AtB‐42 and M10 were inoculated with 9.3 ± 4.4 × 10^4^ and 1.4 ± 0.06 × 10^4^ CFUs·mL^−1^, respectively.

### RNA‐Seq revealed altered protein synthesis and oxidoreductase activities during the in vitro microbe‐microbe interaction

RNA‐seq analysis was conducted on cultures of Experiments A2 at 3 *dfi* to characterize early responses of both strains during their interaction. The confrontation in the liquid co‐cultures induced significant transcriptome changes in both microorganisms. In fact, clearly separated clusters of AtB‐42 and M10 samples were identified by the PCA (Figure S2A,B). Out of 7046 genes in the AtB‐42 transcriptome, 887 were differentially expressed genes (DEGs; false discovery rate or FDR < 0.05) in the co‐culture compared to the single culture, viz. 409 upregulated and 478 downregulated (Figure S2C). Therefore, in AtB‐42, DEGs represented 13% of the transcriptome. In M10 co‐cultures, 1432 DEGs were identified, viz. 706 upregulated and 726 downregulated (Figure S2D). They were 11% of the entire transcriptome (12,842 genes). Genes unaffected by the co‐culturing (non‐regulated genes) were 1726 and 6780 in AtB‐42 and M10, respectively (Figure S2C,D). The remaining genes, that is 4433 of AtB‐42 and 4630 of M10, were not detected in our computational analysis of DEGs very likely because no RNA‐Seq reads mapped to the reference sequences.

**FIGURE 3:**
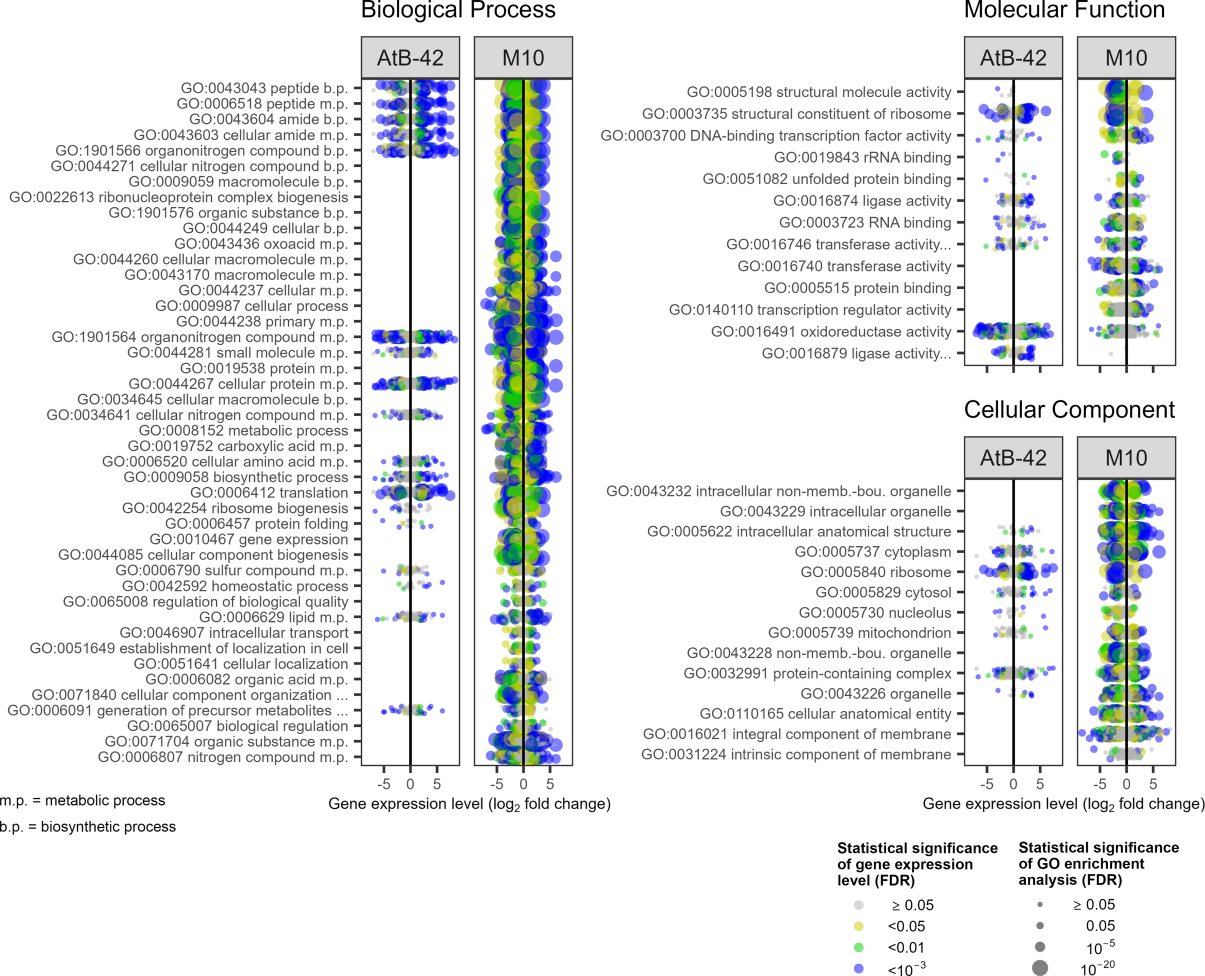
RNA‐Seq analysis of the in vitro liquid co‐culture assays (Experiment A2). Gene expression (log_2_ fold change) of *Streptomyces microflavus* AtB‐42 and *Trichoderma harzianum* M10 in the co‐culture compared to the single cultures. In the graphs, each dot represents the mean (*n* = 3) expression of a gene in the corresponding gene ontology of biological processes, molecular functions or cellular components.

After our genome re‐annotations to get the most updated information, 4299 and 9107 genes were successfully annotated for AtB‐42 and M10, respectively, and thus they were used for the GO analysis (Table S1). A higher number of GOs were found for M10 compared to AtB‐42 (Figure 3), very likely because of a higher amount of information available in the databases. In AtB‐42 co‐cultures, compared to the single cultures, 18 GOs were over‐represented in the biological process (BP) group, 10 in the molecular function (MF) and eight in the cellular component (CC) (Figure 3). In M10, 44, 13 and 14 GOs were over‐represented in BP, MF and CC, respectively. In AtB‐42, enrichment of GOs such as translation (GO:0006412) and peptide biosynthetic process (GO:0043043) were more significant (lower FDR and smaller size of the circles in Figure 3) than others, including lipid (GO:0006629) and sulphur compound metabolic process (GO:0006790), for example. In almost every enriched GOs, the ratios between up‐ and downregulated DEGs were very similar, indicating an overall alteration of those BPs but not their clear induction or suppression.

On the other hand, for AtB‐42, in the organonitrogen compound biosynthetic process (GO:1901566), 119 DEGs were upregulated and 52 were downregulated. In the translation (GO:0006412), 72 DEGs were upregulated and 23 were downregulated. Among the MF GOs, 51 DEGs related to the structural constituent of ribosome (GO:0003735) were upregulated and 10 downregulated. In contrast, 76 DEGs with oxidoreductase activity (GO:0016491) were upregulated and 114 downregulated. Finally, 52 and 11 DEGs in the CC GO ribosome (GO:0005840) were up‐ and downregulated, respectively.

For M10, it was observed a scenario almost specular to that of AtB‐42. The organonitrogen compound biosynthetic process (GO:1901566) and translation (GO:0006412) were altered oppositely compared to AtB‐42: 7 DEGs were upregulated and 76 were downregulated. Similarly, the structural constituent of ribosome (GO:0003735) in the MF group showed three DEGs upregulated and 61 downregulated. On the other hand, oxidoreductase activity (GO:0016491), though over‐represented, did not show a clear induction or suppression as 18 and 27 DEGs were up‐ and down‐regulated, respectively. In the CC group of GOs, the intracellular non‐membrane‐bounding organelle (GO:0043232) and ribosome (GO:0005840) were the most affected GOs with few upregulated DEGs and many downregulated ones.

**FIGURE 4:**
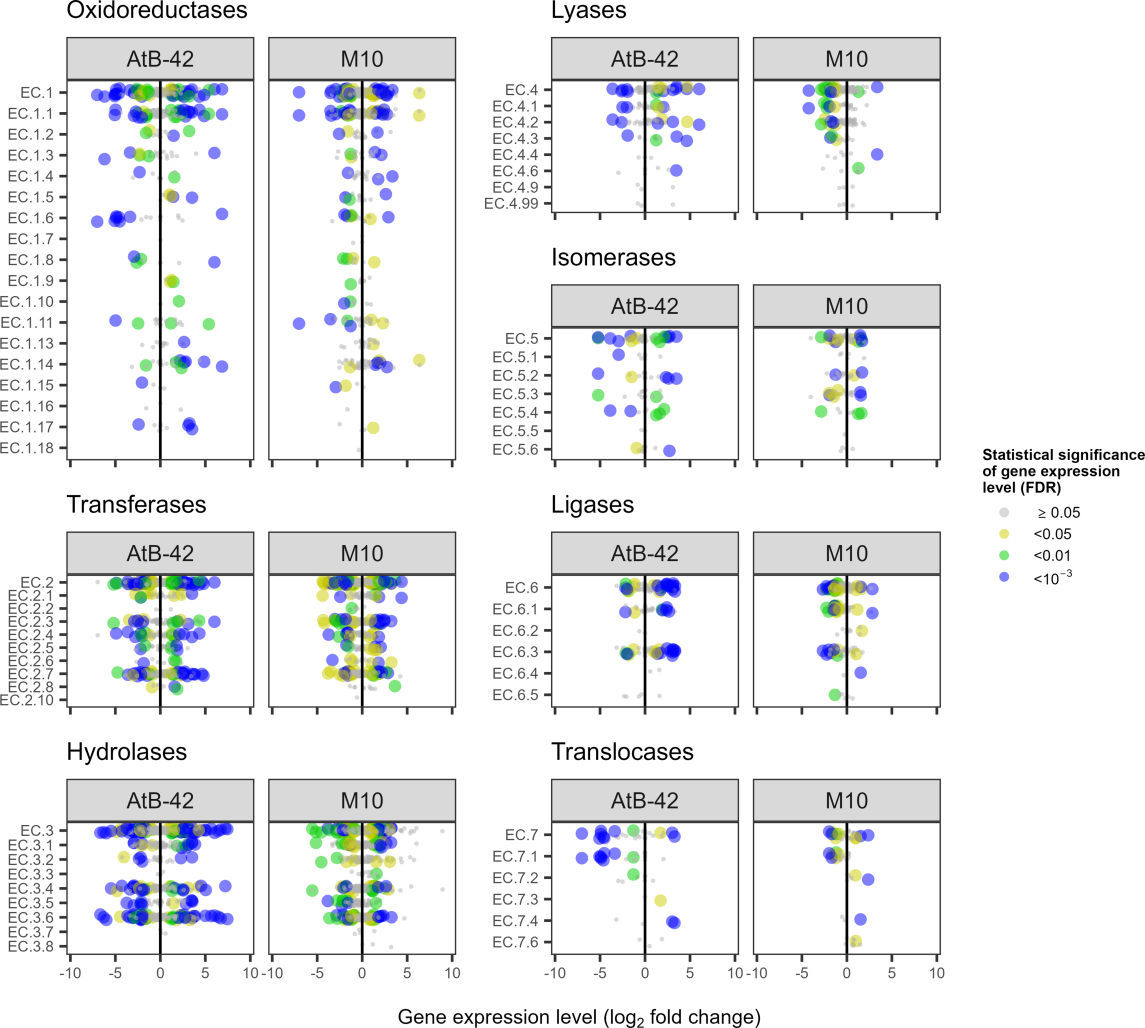
RNA‐Seq analysis of the in vitro liquid co‐culture assays (Experiment A2). Gene expression (log_2_ fold change) of *Streptomyces microflavus* AtB‐42 and *Trichoderma harzianum* M10 in the co‐culture compared to the single cultures. In the graphs, each dot represents the mean (*n* = 3) expression of a gene in the corresponding enzyme family (enzyme commission numbers or EC), which were identified in the genomes of AtB‐42 and M10 using the Blast2GO tool.

Opposite responses of AtB‐42 and M10 also occurred for some enzyme‐coding genes (Figure 4). Most of the DEGs coding for ligases (EC 6) and lyases (EC 4) were upregulated in AtB‐42 and downregulated in M10. Such a contrast also occurred, but to a lesser extent, for the oxidoreductases (EC 1) and hydrolases (EC 3).

**FIGURE 5:**
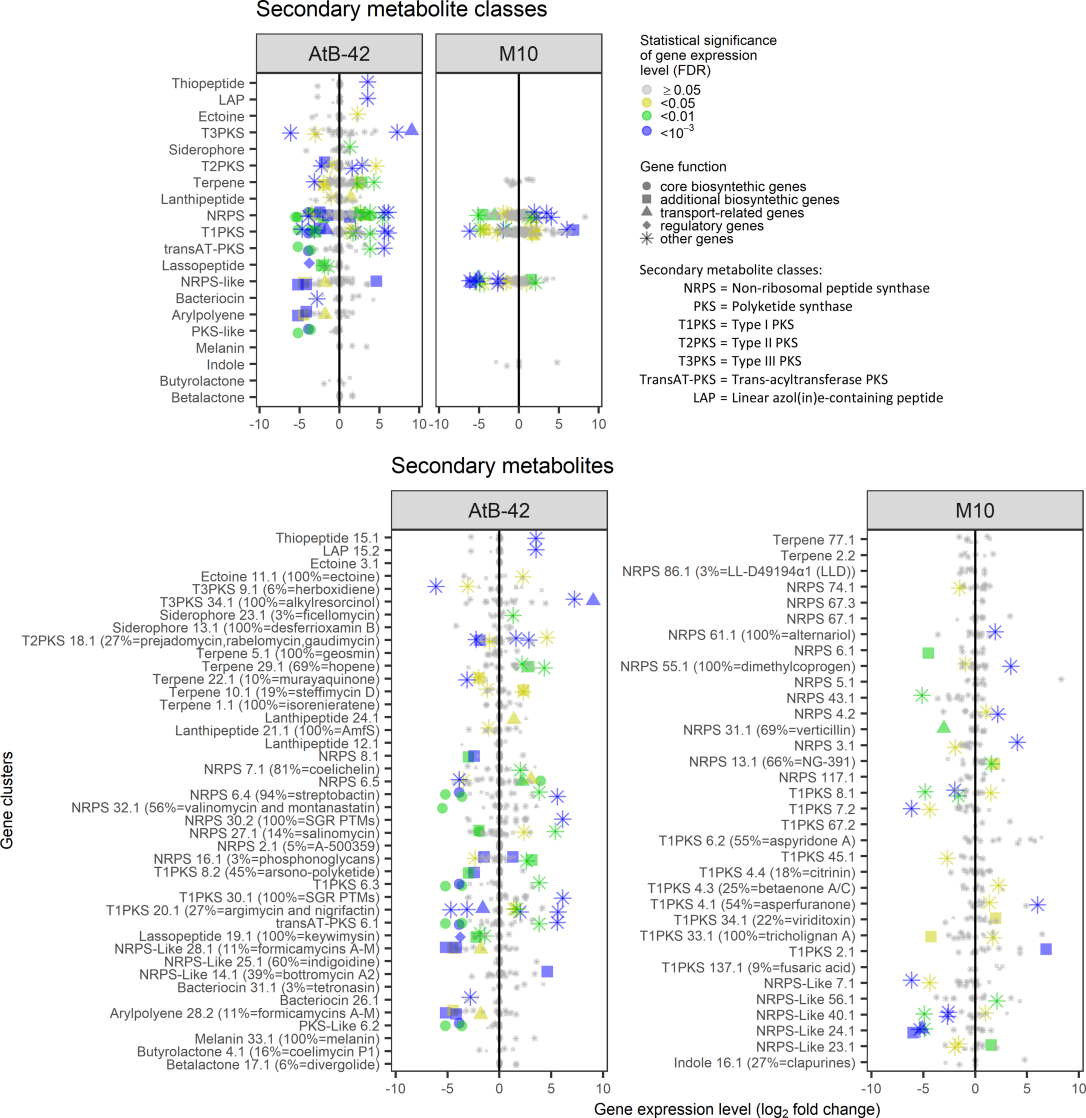
RNA‐Seq analysis of the in vitro liquid co‐culture assays (Experiment A2). Gene expression (log_2_ fold change) of *Streptomyces microflavus* AtB‐42 and *Trichoderma harzianum* M10 in the co‐culture compared to the single cultures. In the graphs, each dot represents the mean (*n* = 3) expression of a gene in the corresponding secondary metabolite class (upper panel) or gene cluster (lower panel), which were identified in the genomes of AtB‐42 and M10 using the antiSMASH tool.

In AtB‐42, 86 out of 1144 genes related to secondary metabolites were differentially regulated in the co‐culture compared to the single culture (Figure 5 and Table S2). In M10, DEGs related to secondary metabolites were 41 out of 409 (Table S2). Among these DEGs (of both strains), only 42 were biosynthetic genes, including 15 genes in the gene cluster core and 27 outside it, while 85 were annotated for transport, regulation or other functions. It is worth mentioning that four AtB‐42's DEGs of the cluster coding for the keywimysin (lassopeptide; region 19.1 as identified by antiSMASH) were all downregulated. Also, three DEGs of a PKS‐like cluster (region 6.2) and four of an arylpolyene cluster (region 28.2, 11% similar with formicamycins A‐M) were downregulated. In M10, several NRPS‐like gene clusters (e.g., regions 7.1, 40.1, 24.1) showed downregulated DEGs.

### In vitro confrontation of AtB‐42 and M10 induces significant changes in the metabolomes of both microorganisms

The in vitro interaction of AtB‐42 and M10 significantly impacted the metabolomes of both strains, as evidenced by the PCA (Figure S3A,B) showing that the co‐cultures clustered separately from the single cultures (controls). A total of 93 compounds produced by AtB‐42 were detected in the co‐culture compared to the single culture. They included 31 differentially accumulated (*p* < 0.05) compounds: nine detected in the co‐culture and 22 detected in the single culture (Table S3). For 23 compounds, the chemical formula was computed based on the molecular mass, including six compounds for which the common name was also identified. In M10, 113 compounds were found, comprising five and 49 detected in the co‐culture and single culture, respectively (Table S4). Chemical formula was deduced for 43 of these compounds. No significantly different accumulation of harzianic acid, isoharzianic acid and harzianolide (i.e. the main metabolites of M10) was detected.

### The consortium reduced the growth of tomato roots, a phenotype not observed upon the single inoculations of AtB‐42 or M10

**FIGURE 6:**
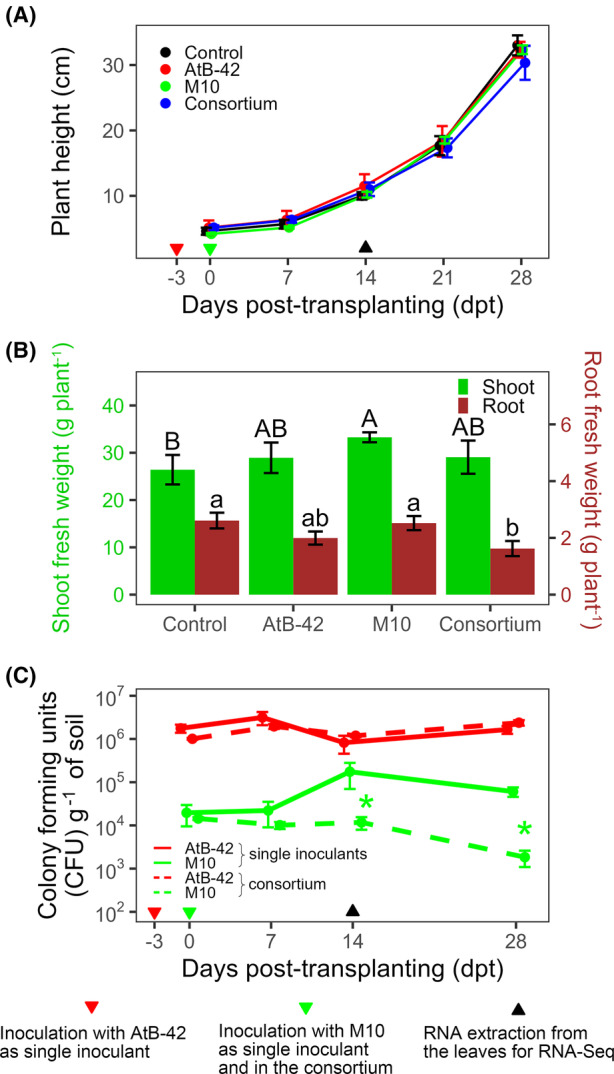
Interaction of *Streptomyces microflavus* AtB‐42 and *Trichoderma harzianum* M10 in the soil (Experiment B1). Effects of the two microbial strains, inoculated separately or in combination (consortium), on the tomato seedlings' development (A and B) and population kinetics in the soil (C). Error bars represent the standard error of the mean (*n* = 3). In A, no significant difference was detected. In B, per each parameter (shoot and root), means with different letters (uppercase and lowercase, respectively) are significantly different according to Fisher's least significant difference (LSD) test (*p* < 0.05). In C, asterisks indicate a significant difference in the consortium compared to the single inoculant according to Student's *t*‐test (*p* < 0.05).

In Experiment B1, the effects of AtB‐42, M10, and the consortium were evaluated on the biomass of tomato seedlings cultivated in pots with sterilized soil. Control plants grew in height from 4.6 ± 0.9 cm at transplanting to 33 ± 2.7 cm at 28 days post‐transplanting (*dpt*; Figure 6A). No significant effects due to microbial inoculations were observed on the plant height over the entire experiment. On the other hand, M10 significantly increased shoot fresh weight by 26%, while the consortium reduced the root fresh weight by 38%, compared to the control (Figure 6B). Actually, AtB‐42 also reduced root weight by 24% compared to the control, though such a reduction was not statistically significant. In the soil, the AtB‐42 population was 1.8 ± 0.7 × 10^6^ CFUs g^−1^ of soil at 0 *dpt* and remained stable over time (1.7 ± 0.06 × 10^6^ CFUs g^−1^ at the end of the experiment; Figure 6C). Its population in the consortium was not statistically different from the single inoculant. M10 was 2 ± 1.7 × 10^4^ CFUs g^−1^ at transplanting and fluctuated between magnitudes of 10^4^ and 10^5^ CFUs g^−1^ over the experiment. In the consortium, M10 kinetics was different from the single inoculation: at 7 and 14 *dpt* the population did not increase compared to 0 *dpt*, while at 14 *dpt* a 10‐fold reduction was observed. At 7 and 14 *dpt*, the M10 population was significantly (*p* < 0.05) less abundant than in the single inoculation treatment.

In another experiment (B2), where the strains were inoculated three times at a 7‐day interval, no significant effects were detected on the plant height (Figure S1A), but the consortium significantly reduced the fresh weight of the shoot and roots (Figure S1B). In a third experiment (B3), significant reductions in the height and normalized vegetation index (NDVI) were observed upon three inoculations with AtB‐42 (Figure S1C,D).

### The microbial consortium induced larger transcriptomic changes in tomato than the single strains

**FIGURE 7:**
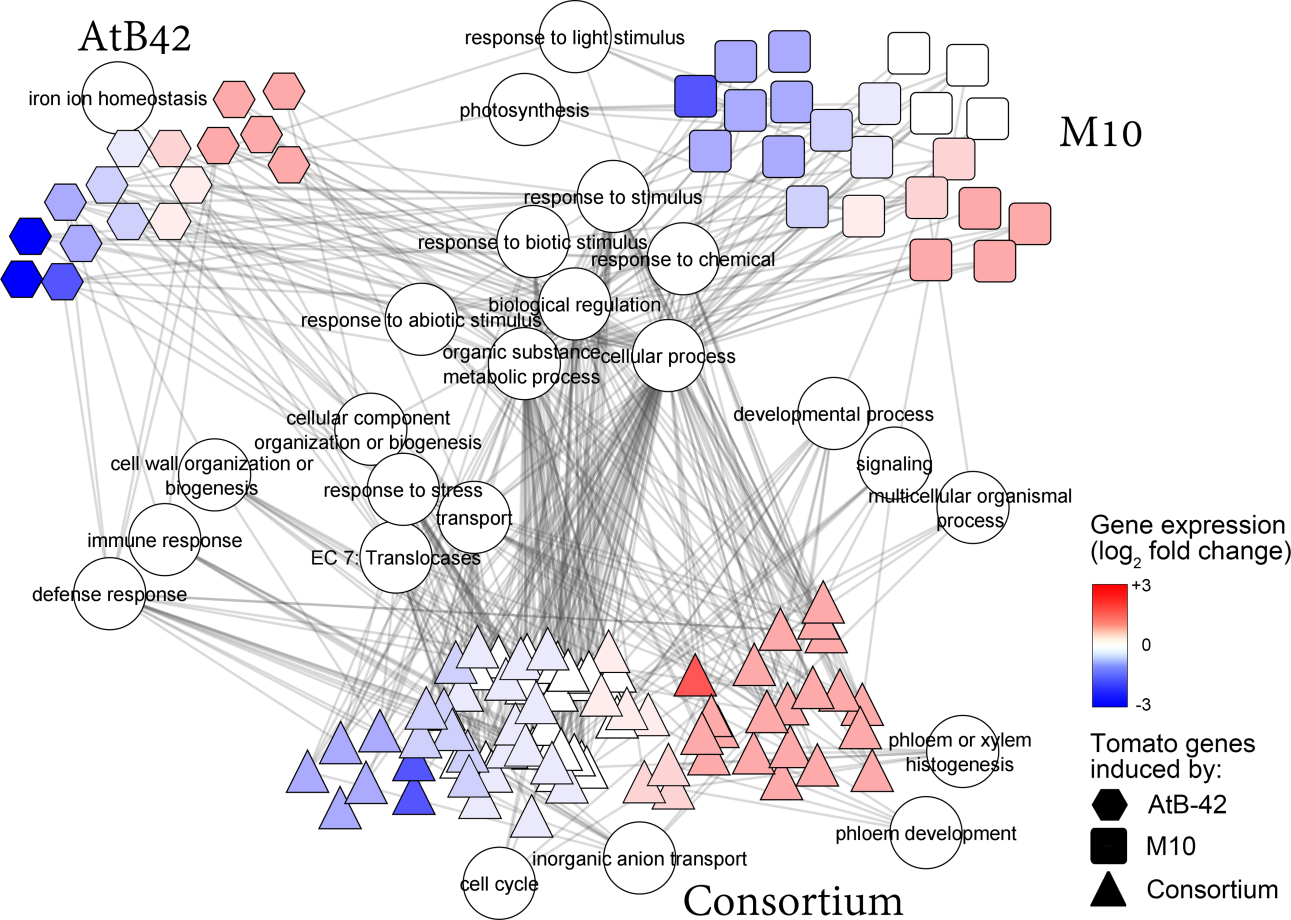
Transcriptome changes of tomato seedlings inoculated with *Streptomyces microflavus* AtB‐42 and *Trichoderma harzianum* M10, separately or in combination (consortium), in the soil (Experiment B1). Network of gene ontologies and associated tomato genes significantly affected (differentially expressed genes or DEGs) by AtB‐42, M10 or the consortium.

The analysis of the transcriptome of tomato plants soil‐drenched with AtB‐42 and M10, separately or in combination (consortium), revealed an overall low number of DEGs. The simultaneous inoculation of both strains (consortium) regulated a greater number of genes compared to their inoculations alone, as shown in Figure S4. Both the Volcano plots and the Eulero‐Venn diagrams showed that among 19,870 detected genes from plants inoculated with the consortium, 96 were upregulated and 65 downregulated with a few transcripts identified in the other inoculation conditions. The network of gene ontologies associated with DEGS showed the main processes affected by the inoculation with AtB‐42, M10 or the consortium (Figure 7). Primary metabolism as well as response to biotic and abiotic stimuli were GOs shared among the three treatments.

Some GOs were affected uniquely by the consortium. Among them, inorganic anion transport was significantly affected as several *Major facilitator superfamily* (*MFS*) *protein* genes (Solyc01g096880, Solyc11g042820 and Solyc03g019650), *Protein NRT1/PTR FAMILY* genes (Solyc11g072580, Solyc06g060620 and Solyc01g080870), and other transporter genes were up‐ or downregulated (Table S5). A *Cellulose synthase* (Solyc09g072820), two *Cyclin* genes (Solyc02g079370, Solyc01g089850), and other few genes, all upregulated, were associated with the cell cycle GO, which was affected only by the consortium. Xylem/phloem histogenesis was another GO peculiar of the consortium. In this GO, several *Sieve element occlusion* genes (Solyc05g013860, Solyc03g111810, Solyc04g026020, Solyc05g013850 and Solyc05g013870) were upregulated. Three *Leucine‐Rich Repeats Receptor‐Like Kinase* (LRR‐RLK) genes (Solyc01g080770, Solyc01g103530 and Solyc05g051640), a *Callose synthase 1* (Solyc07g061920) and other genes were all induced in the signalling and developmental process GOs. In these GOs, two genes were also affected by M10: *Two‐component response regulator ARR9* (Solyc10g079600; upregulated; signalling) and *Gibberellin 20‐oxidase‐2* (Solyc06g035530; downregulated; developmental process). In the response to stress GO, 19 genes were affected by the consortium and six by AtB‐42. LRR‐RLK genes, *Argonaute 10a* (Solyc09g082830), *AP2‐like ethylene‐responsive transcription factor PLT2* (Solyc02g092050), *Glutathione peroxidase* (Solyc08g068800), *Dicer‐like 2d* (Solyc11g008530), and other genes were upregulated by the consortium or AtB‐42, while two chitinases (Solyc10g055780 and Solyc10g055790), two pathogenesis‐related proteins (Solyc08g080620 and Solyc01g097240), and other few genes were downregulated. The consortium also induced many genes devoted to cell wall organization or biogenesis: *Callose synthase 1* (Solyc07g061920), *Cellulose synthase* (Solyc09g072820), *Expansin 2* (Solyc06g049050), *Extensin‐2‐like* (Solyc12g150131) and two *Pectinesterase* genes (Solyc09g075350 and Solyc01g079180).

Genes related to photosynthesis were altered by M10 and AtB‐42. This latter strain induced *K*(+) *efflux antiporter 3* (Solyc11g044250), while M10 downregulated *Photosystem 1 reaction center protein subunit 2* (Solyc06g054260), *Photosystem II reaction center W protein* (Solyc09g065910) and other five genes with the same GO.

## DISCUSSION

The design of a SynCom is challenging because it requires deep knowledge of the interactions among its members and between them and the environment, including other microorganisms, plants and edaphic factors. Understanding such interactions at the community level is moving a step forward (Agler et al., 2016; Toju et al., 2020) to the simpler concept that two or more microbial strains can be combined into a consortium when they are proven to be effective for a given purpose (even if trials are not repeated across seasons and environments; Bradáčová et al., 2019; Minchev et al., 2021; Thakkar & Saraf, 2015). Recently, we experienced that the biocontrol effectiveness can be significantly improved by designing a tailor‐made SynCom compared to a mere mixture of in vitro‐selected antagonists (Prigigallo et al., 2022). Furthermore, consortia with *Streptomyces* spp. and *Trichoderma* spp. have been studied poorly so far. Successful use of *S. rochei* and *T. harzianum* in combination against Phytophthora root rot of pepper is one of the few studies on microbial consortia composed of these two taxa (Ezziyyani et al., 2007). Here, we carried out several experiments to elucidate the interaction between two elite BCAs and to understand whether they are prone to constitute a consortium. Recent promising effects of *S. microflavus* AtB‐42 and *T. afroharzianum* T22 (formerly *T. harzianum* T22; Iacomino et al., 2022; Staropoli et al., 2021) or M10 (Staropoli A. and Vinale F., unpublished) on the qualitative and nutraceutical properties of parsley and tomato in the field have stimulated our further investigation on the interaction between *S. microflavus* strain AtB‐42 and *T. harzianum* strain M10.

In this research, we observed a cross‐inhibition between AtB‐42 and M10 in agar plates due to agar‐diffusible metabolites (DM), produced by both strains and volatile organic compounds (VOCs) emitted by M10 (Figure 1). Moreover, in liquid co‐cultures, M10 promptly compromised the viability of AtB‐42 and was not affected by the presence of the streptomycete (Figure 2). The growth inhibition of AtB‐42 could be due either to metabolites released by M10 and/or scarcity of nutrients after the prompt utilization by the fungus during its rapid growth. Under semi‐controlled conditions like tomato plants grown in pots with sterilized soil, the scenario was inverted: AtB‐42 population kinetics was unaffected by the presence of M10, but M10 growth was partially limited when inoculated in combination with the streptomycete (Figure 6). These in vitro and pot experiments provided some useful information: (a) the success of the consortium applications in the field could not be predicted upon laboratory assays; (b) BCAs compete against each other and the result of such a competition was context‐dependent. It has been known that the interaction between microorganisms is strongly regulated by the extracellular environment (Goers et al., 2014).

The present data confirmed our previous research (Prigigallo et al., 2022) and reinforced our hypothesis that the cross‐inhibition among BCAs is very likely a rule rather than an exception. This could be particularly true in small consortia while, at the community level, it is emerging evidence that fungi stabilize self‐organization and increase the connectivity of multi‐kingdom networks, which also include bacteria, archaea, etc. (Pozo et al., 2021; Yang et al., 2022). The competition between *Trichoderma* spp. and bacteria can occur in nature (Velázquez‐Cedeño et al., 2008), though there have been examples of peaceful coexistence in bioformulations (Comite et al., 2021; Ezziyyani et al., 2007).

In agreement with other studies (Chevrette et al., 2022), RNA‐Seq analysis revealed that the two microorganisms perceived the presence of each other in the in vitro co‐culture (3 days after the co‐inoculation), as evidenced by the reprogramming of both transcriptomes, compared to the single cultures. It could be argued that the transcriptome changes of AtB‐42 (887 DEGs out of 2613 genes) were the result of the growth inhibition due to the presence of M10. Nevertheless, the M10 transcriptome also changed (1432 DEGs out of 8212) even if it grew normally regardless of the presence of the streptomycete. This meant that M10 recognized the presence of AtB42 but its growth remained unaffected.

The growth inhibition of AtB‐42 corresponded to many upregulated genes in GOs such as the organonitrogen compound biosynthetic process (GO:1901566), translation (GO:0006412), structural constituent of ribosome (GO:0003735), oxidoreductase activity (GO:0016491), as well as ligases (EC 6) and lyases (EC 4), suggesting augmented protein synthesis and cellular defence responses very likely activated as an attempt to defend itself from the fungus. Interestingly, in M10, the same GOs were also significantly affected but in the opposite way, viz. with several downregulated genes. In bacteria, ribosome organization has been related to the growth phases (reviewed by Jaishankar & Srivastava, 2017): during the stationary phase, a process termed ribosome hibernation has been thought to fine‐tune the translation (McKay & Portnoy, 2015), and during limited nutrient availability, truncated mRNA and deacylated tRNA accumulate while the ribosomes are trapped on these mRNAs being unable to get released due to the absence of stop codons (Pletnev et al., 2015). In rapidly growing yeast cells, 60% of the whole transcription process is oriented to ribosomal RNA, and 50% of RNA polymerase II transcription events are devoted to ribosomal proteins (Warner, 1999). According to the literature, altered expression of several AtB‐42 genes explained well its growth inhibition. *Developmental transcriptional regulator BldC* (SFUL_RS19000; Bush et al., 2019), *ATP‐dependent Clp protease proteolytic subunit* genes (SFUL_RS10810 and SFUL_RS10815; De Crécy‐Lagard et al., 1999), *DNA‐binding protein WhiA* (SFUL_RS07440) and *WhiB family transcriptional regulator* genes (SFUL_RS13010, SFUL_RS22560 and SFUL_RS23895) have been known to regulate the morphological and physiological differentiation of aerial hyphae in the *Streptomyces* genus (Bush et al., 2015). Also, antibiotic production and resistance were significantly affected because of the impact on genes such as *Developmental transcriptional regulator BldC* (SFUL_RS19000; Bush et al., 2019) *MarR family transcriptional regulator* (SFUL_RS25660; Zhang et al., 2015), *Anti‐sigma factor antagonist* (SFUL_RS15550) and *RNA polymerase sigma factor SigF* genes (SFUL_RS18580 and SFUL_RS13210; Rebets et al., 2018).

Hence, the co‐culture was a stressful condition for both strains and, as such, augmented production of secondary metabolites was expected. It has been documented that *Heterobasidion annosum* produced new metabolites in the presence of antagonistic fungi or plant cells (Sonnenbichler et al., 1989). Likewise, when co‐cultured, probiotic strains of *Lacticaseibacillus casei* and *Lactiplantibacillus plantarum* grew better and produced metabolites not detected in the single cultures (Guo et al., 2022). In our co‐culture experiments, we did not observe a remarkable enhancement of the secondary metabolite production. At the transcriptome level, among 86 DEGs of AtB‐42, 37 were upregulated and 49 were downregulated, while in M10 17 DEGs were upregulated and 24 downregulated (Figure 5). Correspondingly, at the metabolome level, several metabolites were uniquely detected in the co‐cultures or the single cultures (Tables S3 and S4). In AtB‐42, for example, the co‐culturing induced the production of glucopiericidin and hampered that of cyclipostin, ripromycin, WK 142B, delaminomycin A and Formycin A, which are well‐known antibiotics of the *Streptomyces* genus (Azad et al., 2022; Bertasso et al., 2003; Hori et al., 1964; Omura et al., 1986; Ueno et al., 1993; Wink et al., 2002). It should be mentioned that, besides the huge literature on the co‐cultures of microorganisms, few articles report the enhancement of the overall production of metabolites. More than anything else, the co‐culturing of microbes has been proposed as a strategy to activate the silent gene clusters and thus obtain new bioactive molecules (Peng et al., 2021). This has been demonstrated for several microorganisms including *T. harzianum* M10: when this strain was co‐cultured with *Talaromyces pinophilus*, a new metabolite was discovered, that is the harziaphilic acid, though very few other metabolites were accumulated differentially (Vinale et al., 2017). Interestingly, it has also been proven that plant pathogens can stimulate the antimicrobial activity of BCAs by improving the production of specific secondary metabolites and the transcription of genes related to their precursors. In the *Trichoderma*‐*Botrytis* interaction, sesquiterpenes produced by the pathogen triggered the production of polyketides by *T. arundinaceum* (Malmierca et al., 2015) and polyketides produced by *Botrytis cinerea* induced the expression of genes involved in sesquiterpene biosynthesis in *T. arundinaceum* (Malmierca et al., 2016). Similarly, in the *Streptomyces*‐*Magnaporthe* interaction, *NRPS* gene expression levels and lipopeptide production of *S. bikiniensis* were significantly augmented in vitro in the presence of *M. oryzae* (Liu et al., 2022).

In planta (pot experiments), the consortium of AtB‐42 and M10 significantly reduced the root weight of tomato seedlings by 38% compared to untreated plants, M10 increased the shoot weight by 26%, while AtB‐42 alone did not affect significantly the plant growth (Figure 6B). In supplemental pot experiments under different conditions, AtB‐42 alone or in combination with M10 reduced the shoot weight, root weight or plant height (Figure S1). Collectively, based on these pot experiments, it could be argued that AtB‐42 tended to induce growth stunting in tomato plants and contrast the plant growth promoted by M10. However, it is worth mentioning that AtB‐42 has increased the biomass and yield of tomatoes in pot experiments (applied along with compost; Colella et al., 2001) as well as the biomass and nutraceutical value of parsley in the field (Staropoli et al., 2021). It is widely known that the applications of microorganisms in agriculture provide variable results across different environments. Also, the inoculum concentration of BCAs has been highlighted as an important factor in modulating plant growth. Excessive concentrations of *T. asperellum* (10^6^–10^8^ conidia mL^−1^) for bio‐priming seeds of six vegetable crops have resulted in reductions in radicle length and seed germination (Singh et al., 2016).

RNA‐Seq revealed significant leaf transcriptome reprogramming upon inoculation with the microorganisms in the soil (Figure S4); hence, a systemic signal migrated from the roots interacting with the microorganisms to the shoot. Overall, the consortium had a greater effect on the transcriptome compared to the single inoculants, as it significantly impacted more genes (Figure S4B,C). The few DEGs regulated by the single inoculants could suggest that a very early response was captured with the RNA‐Seq performed at 2 weeks post‐transplanting tomato seedlings into inoculated soil, and more time would be required to get stronger transcriptome changes (more DEGs) with our BCAs. Often, hundreds of DEGs have been detected in RNA‐Seq experiments with single BCAs (Vergnes et al., 2019; Wu et al., 2018; Zhao et al., 2023), while we observed few tens of DEGs upon inoculations with AtB‐42 or M10 alone, a case that may also occur (Perazzolli et al., 2012). The treatments with either the single strains or the consortium impacted almost the same GOs, including primary metabolism, biological, catabolic and cellular processes, photosynthesis, responses to stimuli, chemicals, abiotic and biotic stresses, signalling, transmembrane transport and a few others (Figure 7). Hormone metabolism, response to stimuli, photosynthesis, ion transport and other processes have been affected frequently in plants inoculated with BCAs (De Palma et al., 2019, 2021; Kruasuwan et al., 2023; Wu et al., 2018; Zhao et al., 2023), though certain pathways have been more strain‐specific (Weston et al., 2012).

We postulated that a fitness cost due to the activation of defence to stresses by our BCAs could result in plant growth stunting. Tomato plants inoculated with AtB‐42, compared with the control, showed regulation, positive or negative, of several genes related to the response to biotic or abiotic stimuli. Among the upregulated genes, Dicer‐like genes such as *Dicer‐like 2d* (Solyc11g008530) have been known to be activated under viral infection and various abiotic stresses (Bai et al., 2012). *NAC domain‐containing protein 10* (Solyc02g093420) is an orthologous gene of SUPPRESSOR OF GAMMA RESPONSE 1 (SOG1), which in Arabidopsis is a key transcription factor promoting DNA damage repair and targeting several defence‐related genes such as those required for resistance against the hemi‐biotrophic fungus *Colletotrichum higginsianum* (Ogita et al., 2018). ATP‐binding cassette (ABC) transporter gene superfamily like *ABC transporter B family member 27* (Solyc03g114950) governs herbicide and xenobiotic resistance in plants (e.g., cadmium, lead) as well as cancer resistance in humans and drug resistance among vertebrates (Lane et al., 2016). *Peroxidase* (Solyc11g018774), *PR5‐x* (Solyc08g080620) and *Chitinase* (Solyc10g055780) were downregulated. These genes are involved in phenylpropanoid/lignin biosynthesis, which is one of the processes of systemic acquired resistance (SAR). More typically, BCAs activate the induced systemic resistance (ISR), which is in antagonism with SAR (Vallad & Goodman, 2004). Nevertheless, the activation of SAR (e.g., phenylpropanoids, pathogenesis‐related or PR proteins, chitinases, etc.) or both SAR and ISR, by BCAs has also been known (Malmierca et al., 2012; Nawrocka & Małolepsza, 2013; Salas‐Marina et al., 2011). More than one report has documented that the resistance induction by *Streptomyces* spp. occurred by both SAR and ISR pathways (Conn et al., 2008; Kurth et al., 2014; Vergnes et al., 2019) and several plant hormones may be affected (Belt et al., 2021; Kruasuwan et al., 2023; Wu et al., 2018). The downregulation of *Peroxidase* (Solyc11g018774), *PR5‐x* (Solyc08g080620) and *Chitinase* (Solyc10g055780) would be consistent with the activation of ISR by AtB‐42. In several other experiments with streptomycetes, the alteration of the metabolisms of plant hormones such as abscisic, salicylic and jasmonic acids has been observed (Belt et al., 2021; Kruasuwan et al., 2023; Kurth et al., 2014; Vergnes et al., 2019; Wu et al., 2018).

In M10‐treated plants, some upregulated genes were related to primary metabolic processes, very likely underlying the plant growth promotion observed. The impact of M10 on tomato growth was also suggested by the differential regulation of *gibberellin 20‐oxidase‐2* (Solyc06g035530) and *response regulator 6* (Solyc10g079600), which is probably a negative regulator of the cytokinin signalling (Fleishon et al., 2011). Nevertheless, several genes related to photosynthesis and the response to light stimuli were downregulated, except the *nuclear transcription factor Y subunit A‐3* (Solyc12g009050), which has been known to modulate the expression of photosynthetic genes as well as plant growth and stress responses (Zhao et al., 2017). This was not in agreement with previous research documenting the enhancement of photosynthetic molecular processes upon applications of different beneficial *Trichoderma* spp. strains in tomatoes and other plants (Coppola et al., 2019; De Palma et al., 2021; Doni et al., 2019; Manganiello et al., 2018; Shoresh & Harman, 2008). However, it should be noted that the depletion of photosynthesis‐related genes or host plant carbon gain have been observed during the induction of systemic defence responses by *Streptomyces* sp. or *Pseudomonas fluorescens* strains, and this phenomenon was interpreted as a kind of fitness cost (Kurth et al., 2014; Weston et al., 2012). Perazzolli et al. (2012) experienced that only a few genes were affected by the inoculation of grapevine with *T. harzianum* T39. Plant defence responses and microbe recognition were the two processes mainly affected, suggesting that they could be among the first mechanisms activated in that plant‐microbe interaction. In another research, *T. afroharzianum* T22 triggered many genes in tomato roots at the onset of the interaction (i.e. 24, 48 and 72 h post‐inoculation), and they were mainly ascribable to signal recognition, transduction, stress response, transcriptional regulation and transport (De Palma et al., 2019). We also observed a substantially similar scenario: primary metabolism, signal transduction, transmembrane transport (likely involved in microbe recognition by the plant), and defence responses were probably detected because they represented the early host response to M10.

Our *T. harzianum* strain also variably regulated myeloblastosis (MYB) and basic helix–loop–helix (bHLH) transcription factors (TFs; Solyc02g084880, Solyc06g062460 and Solyc01g108300), which have multiple functions in plant growth, stress response, secondary metabolism and hormone signal transduction (Cao et al., 2020; Hao et al., 2021; Katiyar et al., 2012). Significant modulation of TFs, especially of the MADS‐box, MYB, WRKY, NAC and ERF families has also been observed by De Palma et al. (2021) upon inoculation of tomatoes with *T. longibrachiatum*. In that research, many TFs involved in plant development were mainly downregulated. Significant effects on MYB and NAC TFs, along with ethylene metabolism, have also been observed in *T. harzianum*‐grapevine interaction (Perazzolli et al., 2012).

In the plants inoculated with our consortium, some TFs, including one bHLH TF and two ethylene‐responsive TFs (Solyc02g091690, Solyc08g081960 and Solyc02g092050), as well as other genes involved in the signal transduction like Leucine‐Rich Repeats Receptor‐Like Kinases (LRR‐RLKs) and histidine kinases were also induced. In the signal transduction GO, the *hydroxyproline‐rich systemin* (Solyc06g068520) was activated, suggesting that BCAs can also induce the smallest hormone of the plant kingdom. Systemin has been thought to protect from herbivore and pathogen attacks (Bhattacharya et al., 2013; Molisso et al., 2021; Zhang et al., 2020). The increased expression of *AP2‐like ethylene‐responsive transcription factor PLT2* (Solyc02g092050) and *Solanum lycopersicum Cytokinin Response Factor 2* (Solyc08g081960) along with the downregulation of *Chitinase* (Solyc10g055790), *PR‐4* (Solyc01g097240) and a *Major latex‐like protein* [MLP‐like; Solyc04g007790; MLPs induce PR proteins (Fujita & Inui, 2021)], indicated that the consortium would activate the defence responses via the ethylene/jasmonate pathway (ISR), which was in agreement with other research works conducted with *T. harzianum* strains (De Palma et al., 2019; Manganiello et al., 2018). Nevertheless, the upregulation of another MLP‐like protein gene (Solyc05g005865), the diverse modulation of several NRT1/PTR FAMILY proteins (Solyc11g072580, Solyc06g060620, Solyc01g080870), which transport plant hormones and secondary metabolites (Chiba et al., 2015), and the induction of a gene responding to auxins (Solyc04g081270), which have been found regulated by rhizobacteria, streptomycetes and *T. harzianum* (Belt et al., 2021; De Palma et al., 2019; Zhang et al., 2007), would underlie that a crosstalk among several hormones occurred in tomato plants inoculated with the consortium.

Some altered GOs were peculiar only to the consortium‐inoculated plants. DEGs involved in the cell cycle like cyclins (Solyc02g079370 and Solyc01g089850) and *Cellulose synthase* (Solyc09g072820) were upregulated. Genes participating in the transmembrane transport were mostly upregulated: major facilitator superfamily proteins (Solyc03g019650, Solyc01g096880 and Solyc11g042820), NRT1/PTR FAMILY proteins (Solyc11g072580, Solyc06g060620, Solyc01g080870), transporters of molybdate, sugar and HCO_3_^−^ (Solyc03g119930, Solyc02g079220 and Solyc01g079150). Transcriptional regulation of plant sugar transporters (Solyc02g079220) and aquaporins (Solyc01g111660) has been already documented for plant‐beneficial rhizobacteria or arbuscular mycorrhizae (Desrut et al., 2021; Wang et al., 2018). Phloem/xylem histogenesis and development were also impacted by the consortium, with the upregulation of five *Sieve element occlusion* genes (Solyc04g026020, Solyc05g013860, Solyc03g111810, Solyc05g013870, Solyc05g013850), which encode structural phloem proteins involved in phloem sealing after wounding by sap‐feeding insects, with a role that is still debated (Ernst et al., 2012; Knoblauch et al., 2014; Kondo et al., 2016). *Cellulose synthase* (Solyc09g072820), required for xylem development (Daras et al., 2021) and *Callose synthase 1* (Solyc07g061920), which exercises important functions during development and stress responses (Alonso‐Ramirez et al., 2014; Wang et al., 2021, 2022), were both induced. So far, this process and these genes have been poorly reported to be involved in the interactions between plants and beneficial microorganisms. Callose accumulation occurring in salicylic acid‐dependent defence responses has been related to the hardening of plant cell walls for the confinement of *Trichoderma* spp. in the apoplast (Morán‐Diez et al., 2021).

## CONCLUSIONS

We investigated in detail the interaction between two strains of *S. microflavus* and *T. harzianum* in an attempt to define a workflow for the construction of a microbial consortium. The laboratory assays (in plates and sterilized soil) were not fully informative about the compatibility of the microorganisms because extremely context‐dependent. From this and our previous research (Prigigallo et al., 2022), it was evident that microorganisms, including BCAs, compete against every other microorganism to preserve the species and their niche. Previously, we were successful to optimize a SynCom based only on in vitro assays (Prigigallo et al., 2022). Nevertheless, here we show that laboratory experiments could be helpful only partially to predict the performance of the microorganisms in the field because in vitro and in vivo experiments do not necessarily correlate. Whenever possible, hence, it would be more advantageous to study the microorganisms in vivo. Finally, the analysis of the plant transcriptome represented a valuable tool to interpret the phenotypic effects of the BCAs on the crop.

## AUTHOR CONTRIBUTIONS

**Maria Isabella Prigigallo:** Conceptualization (equal); formal analysis (equal); investigation (equal); methodology (equal); validation (equal); writing – original draft (equal); writing – review and editing (equal). **Alessia Staropoli:** Formal analysis (supporting); investigation (supporting); methodology (supporting); writing – review and editing (supporting). **Francesco Vinale:** Funding acquisition (equal); investigation (supporting); methodology (supporting); writing – review and editing (supporting). **Giovanni Bubici:** Conceptualization (equal); data curation (equal); funding acquisition (equal); investigation (equal); methodology (equal); supervision (equal); visualization (equal); writing – original draft (equal); writing – review and editing (equal).

## CONFLICT OF INTEREST STATEMENT

The authors declare no conflict of interest.

## Supporting information


Appendix S1

## Data Availability

RNA‐Seq data of the in vitro and in planta experiments are available with the BioProject IDs PRJNA954200 and PRJNA954272, respectively.
