# Supplementary material for: Interactions between plant‐beneficial microorganisms in a consortium: Streptomyces microflavus and Trichoderma harzianum
Source: Microb Biotechnol. 2023 Jul 18;16(12):2292–312. doi: 10.1111/1751-7915.14311 (PMC10686133; doi:10.1111/1751-7915.14311)
Supplement: Supplementary file 1 — Appendix S1 [file MBT2-16-2292-s001.zip › Manuscript 2 Supporting Information.docx]

**SUPPORTING INFORMATION**

for the article

**Interactions between plant-beneficial microorganisms in a consortium: *Streptomyces microflavus* and *Trichoderma* *harzianum***

Maria Isabella Prigigallo, Alessia Staropoli, Francesco Vinale, Giovanni Bubici

**Table S1.** Gene expression (RNA-Seq) by *Streptomyces microflavus* AtB-42 and *Trichoderma harzianum* M10 in the *in vitro* single- and co-cultures.

**Table S2.** Expression (RNA-Seq) of genes related to secondary metabolites in *Streptomyces microflavus* AtB-42 and *Trichoderma harzianum* M10 in the *in vitro* single- and co-cultures.

**Table S3.** Metabolites differentially produced by *Streptomyces microflavus* AtB-42 in the single culture and in the co-culture with *Trichoderma harzianum* M10.

| Compounds | Monoisotopic mass (Da) | Retention time (min) |
| --- | --- | --- |
| *Detected in the co-culture:* |  |  |
| Glucopiericidin A/B | 577.3654 | 4.797 |
| C_7_H_7_NO_2_ | 137.0478 | 0.762 |
| C_13_H_16_O_2_ | 204.1152 | 5.869 |
| C_10_H_13_N_5_ | 203.1156 | 0.799 |
| C_28_H_55_N_5_O_10_ | 621.3916 | 4.869 |
| n.c. | 783.4832 | 4.797 |
| n.c. | 797.4607 | 4.847 |
| n.c. | 841.4874 | 4.899 |
| n.c. | 858.5144 | 4.896 |
| *Detected in the single culture:* |  |  |
| Formycin A | 267.0954 | 0.810 |
| Cyclipostin Q/Q3/S | 458.2750 | 4.834 |
| Delaminomycin A | 501.3128 | 0.837 |
| Ripromycin | 524.2797 | 0.843 |
| WK 142B | 753.4346 | 1.003 |
| C_17_H_37_NO_2_ | 287.2832 | 5.920 |
| C_17_H_37_NO_2_ | 287.2833 | 5.704 |
| C_28_H_43_NS_2_ | 457.2866 | 0.837 |
| C_20_H_43_N_11_O_6_ | 533.3394 | 1.056 |
| C_27_H_45_N_8_O_2_S | 545.3387 | 0.837 |
| C_25_H_43_N_12_O_3_ | 559.3576 | 5.119 |
| C_29_H_49_N_8_O_3_S | 589.3655 | 0.840 |
| C_19_H_48_N_8_O_13_S | 628.3067 | 0.873 |
| C_31_H_53_N_8_O_4_S | 633.3908 | 0.843 |
| C_30_H_59_N_5_O_11_ | 665.4203 | 4.721 |
| C_24_H_47_N_21_O_3_ | 677.4178 | 0.865 |
| C_22_H_50_N_15_O_10_S | 716.3589 | 0.906 |
| C_29_H_61_N_11_O_11_ | 739.4550 | 0.939 |
| n.c. | 973.5690 | 4.904 |
| n.c. | 1017.595 | 4.954 |
| n.c. | 1034.623 | 4.954 |
| n.c. | 1122.675 | 5.031 |

*n.c. = molecular formula not computed.*

**Table S4.** Metabolites differentially produced by *Trichoderma harzianum* M10 in the single culture and in the co-culture with *Streptomyces microflavus* AtB-42.

| Compound | Monoisotopic mass (Da) | Retention time (min) |
| --- | --- | --- |
| *Detected in the co-culture:* |  |  |
| C_13_H_17_N_5_O_2_ | 275.1376 | 0.828 |
| C_26_H_51_N_5_O_9_ | 577.3654 | 4.797 |
| C_26_H_52_N_6_O_12_S | 672.3325 | 0.949 |
| n.c. | 783.4832 | 4.797 |
| n.c. | 797.4607 | 4.847 |
| *Detected in the single culture:* |  |  |
| C_16_H_34_O_4_ | 290.2463 | 7.361 |
| C_10_H_20_N_6_O_5_ | 304.1484 | 0.841 |
| C_17_H_20_O_6_ | 320.1220 | 0.825 |
| C_13_H_24_N_7_O_3_ | 326.1942 | 1.214 |
| C_11_H_21_N_11_O_2_ | 339.1896 | 1.040 |
| C_14_H_26_N_3_O_7_ | 348.1743 | 0.838 |
| C_11_H_26_N_2_O_10_S | 378.1276 | 0.829 |
| C_21_H_29_N_5_S | 383.2161 | 1.116 |
| C_15_H_31_N_8_O_4_ | 387.2470 | 1.214 |
| C_16_H_33_N_8_O_4_ | 401.2622 | 0.938 |
| C_16_H_2_N_14_ | 414.2460 | 0.844 |
| C_16_H_26_N_2_O_11_ | 422.1532 | 0.827 |
| C_17_H_35_N_8_O_5_ | 431.2731 | 1.208 |
| C_17_H_33_N_8_O_6_ | 445.2517 | 0.843 |
| C_19_H_26_N_6_O_8_ | 466.1794 | 0.844 |
| C_19_H_39_N_8_O_6_ | 475.2988 | 1.210 |
| C_22_H_44_N_6_O_6_ | 488.3329 | 7.300 |
| C_19_H_45_N_4_O_10_ | 489.3143 | 0.994 |
| C_23_H_32_N_3_O_10_ | 510.2049 | 0.864 |
| C_22_H_45_NO_12_ | 515.2947 | 4.698 |
| C_21_H_43_N_8_O_7_ | 519.3259 | 0.860 |
| C_22_H_42_N_3_O_11_ | 524.2775 | 0.860 |
| C_25_H_51_N_8_O_4_ | 527.4037 | 7.283 |
| C_21_H_41_N_8_O_8_ | 533.3046 | 0.905 |
| C_20_H_43_N_11_O_6_ | 533.3404 | 1.075 |
| C_28_H_34_N_4_O_8_ | 554.2313 | 0.869 |
| C_27_H_46_NO_11_ | 560.3010 | 0.882 |
| C_24_H_46_N_3_O_12_ | 568.3031 | 0.864 |
| C_27_H_55_N_8_O_5_ | 571.4299 | 7.267 |
| C_23_H_45_N_8_O_9_ | 577.3307 | 0.907 |
| C_27_H_48_N_4_O_11_ | 604.3269 | 0.899 |
| C_24_H_45_N_15_O_4_ | 607.3786 | 0.886 |
| C_28_H_48_N_11_O_6_ | 634.3739 | 0.891 |
| C_27_H_55_N_8_O_10_ | 651.4049 | 0.899 |
| C_28_H_54_N_3_O_14_ | 656.3550 | 0.893 |
| C_27_H_53_N_8_O_11_ | 665.3834 | 0.932 |
| C_28_H_57_N_8_O_10_ | 665.4199 | 4.753 |
| C_31_H_58_N_4_O_12_ | 678.3995 | 0.913 |
| C_29_H_57_N_8_O_12_ | 709.4083 | 0.964 |
| C_31_H_63_N_8_O_12_ | 739.4558 | 0.950 |
| n.c. | 753.4346 | 1.019 |
| n.c. | 783.4813 | 1.000 |
| n.c. | 960.5517 | 4.924 |
| n.c. | 973.5682 | 4.913 |
| n.c. | 1004.578 | 4.968 |
| n.c. | 1017.594 | 4.963 |
| n.c. | 1034.623 | 4.957 |
| n.c. | 1048.603 | 5.013 |
| n.c. | 1122.685 | 5.035 |

*n.c. = molecular formula not computed.*

**Table S5.** Gene expression (RNA-Seq) in tomato plants inoculated with *Streptomyces microflavus* AtB-42, *Trichoderma harzianum* M10, or a consortium composed of these two strains.


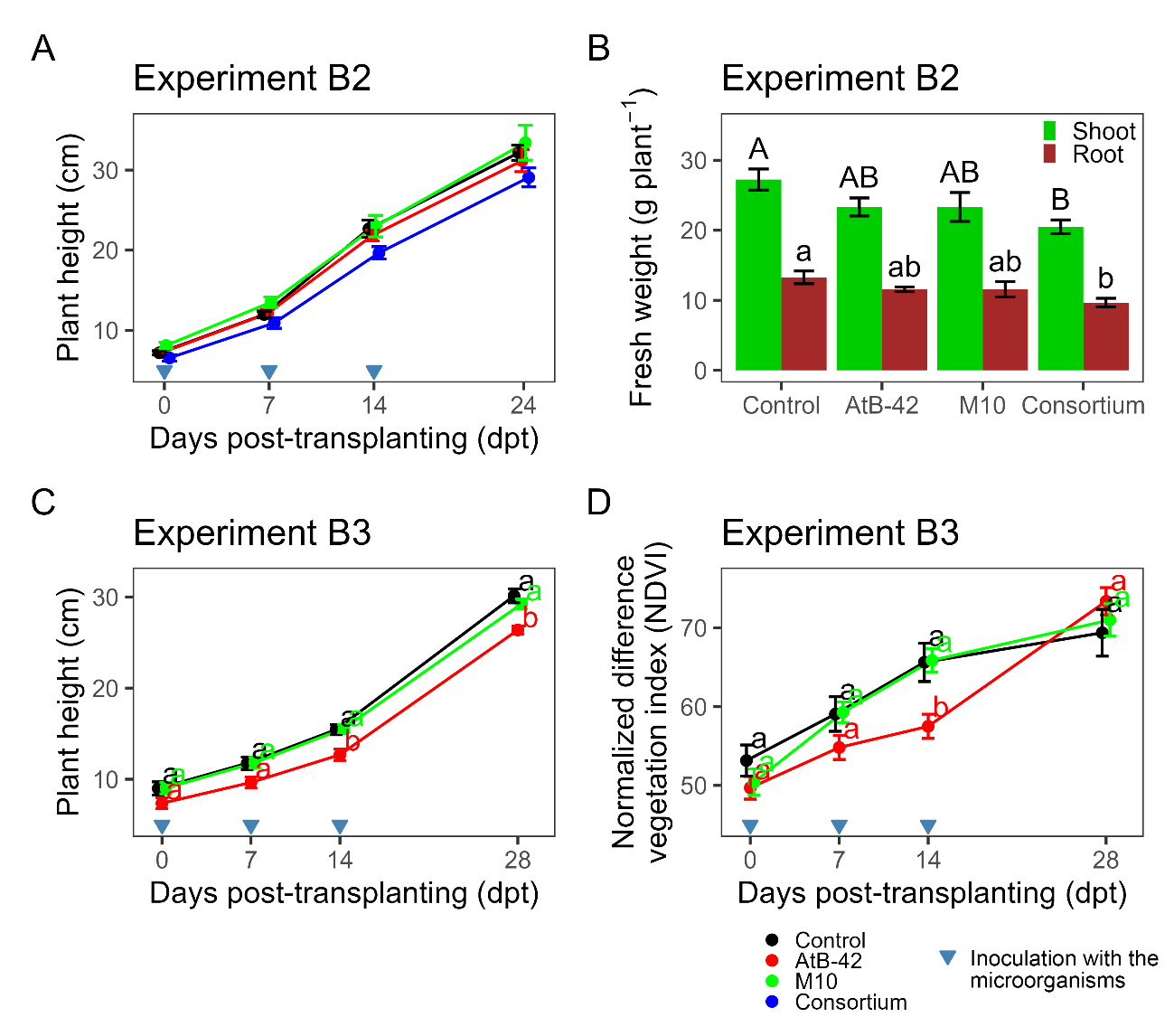


**Figure S1.** Interaction of *Streptomyces microflavus* AtB-42 and *Trichoderma harzianum* M10 in the soil (Experiment B1). Effects of the two microbial strains, inoculated separately or in combination (consortium), on the tomato seedlings’ development in two independent experiments (B2 and B3). Error bars represent the standard error of the mean (n=4). Means with different letters (uppercase or lowercase) are significantly different according to Fisher’s least significant difference (LSD) test (*P*<0.05). In A, no statistically significant difference was detected. In B, the LSD test was performed separately per each parameter (shoot and root).


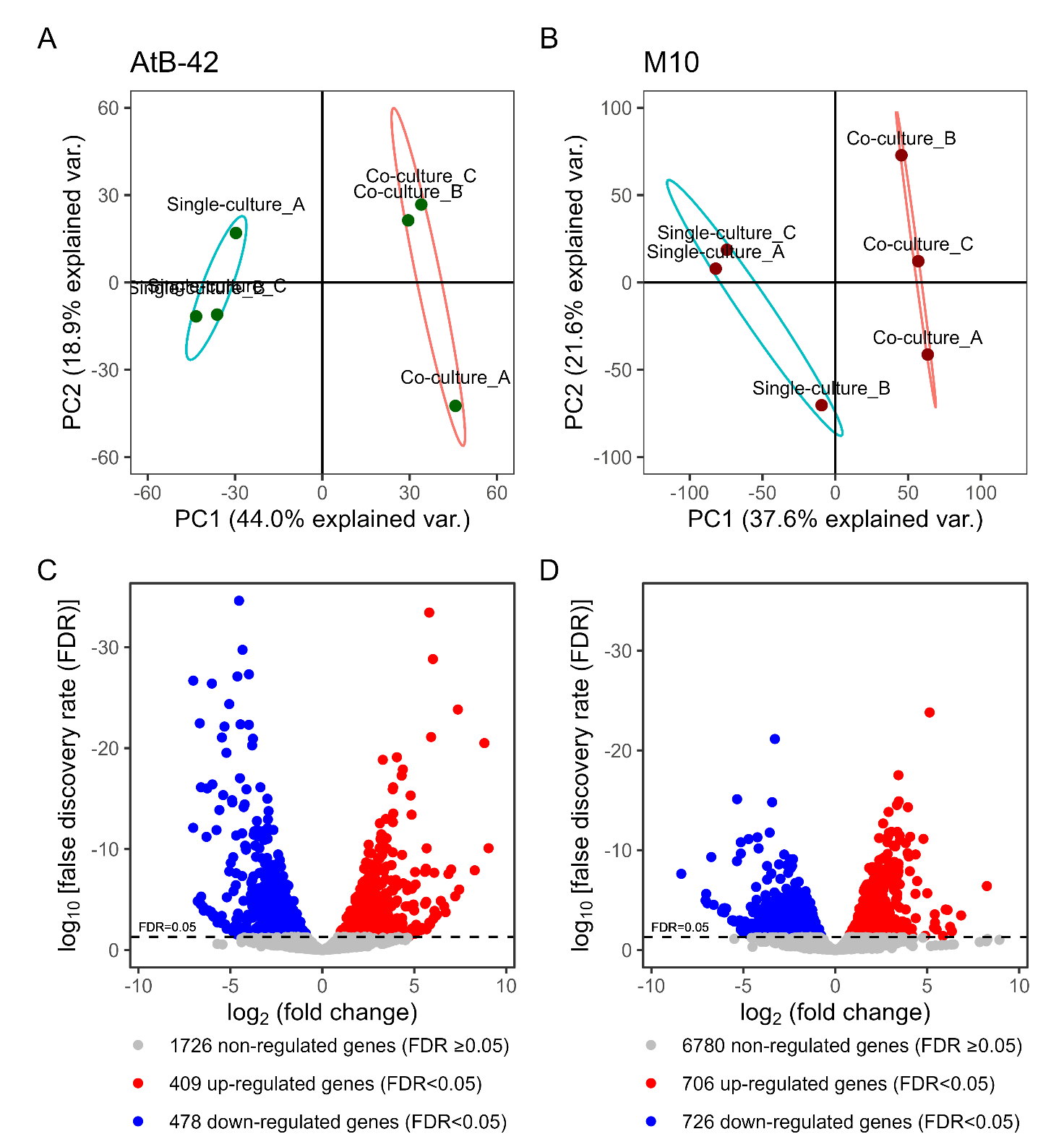


**Figure S2.** RNA-Seq analysis of the *in vitro* liquid co-culture assays (Experiment A2). Principal component analysis (PCA; A and B) and volcano plots (C and D) of the transcriptome changes of *Streptomyces microflavus* AtB-42 (A and C) and *Trichoderma harzianum* M10 (B and D) in the co-culture compared to the single cultures.


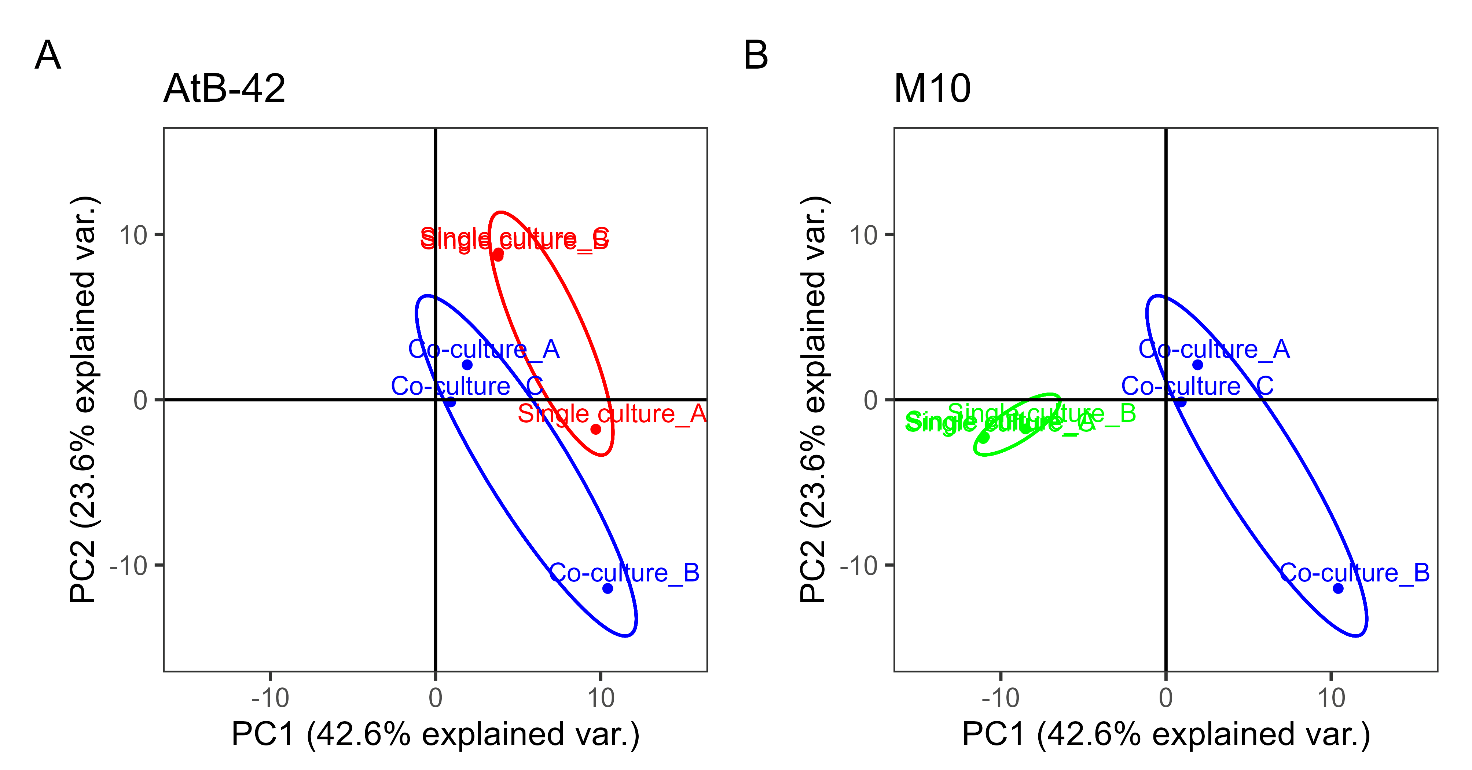


**Figure S3.** Metabolomic analysis of the *in vitro* liquid co-culture assays (Experiment A2). Principal component analysis (PCA) of the metabolome changes of *Streptomyces microflavus* AtB-42 (A) and *Trichoderma harzianum* M10 (B) in the co-culture compared to the single cultures.


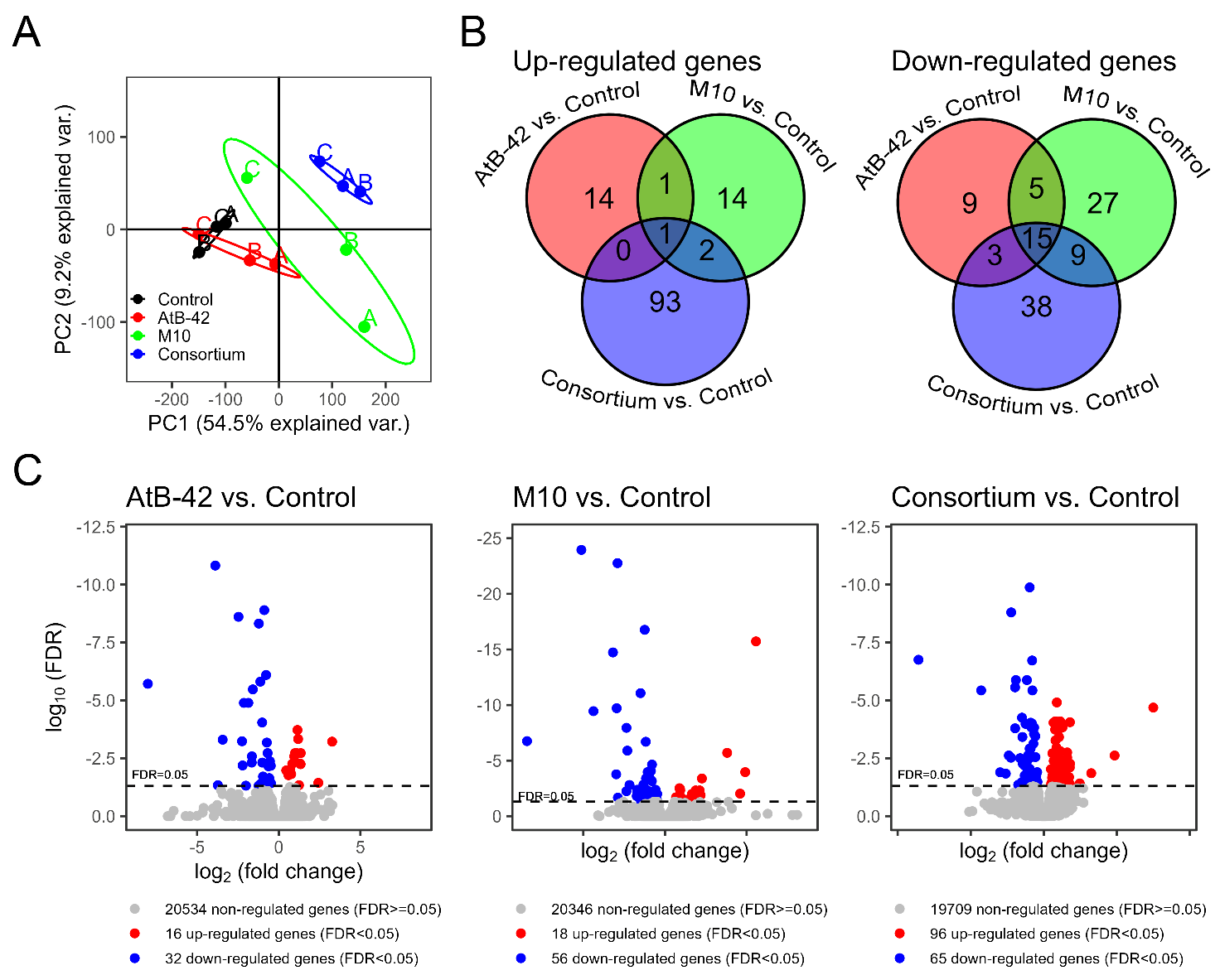


**Figure S4.** Transcriptome change of tomato seedlings inoculated with *Streptomyces microflavus* AtB-42 and *Trichoderma harzianum* M10, separately or in combination (consortium), in the soil (Experiment B1). Principal component analysis (PCA; A), Eulero-Venn diagrams (B), and volcano plots (B).
